# The recognition method of external force damage sources vibration signals based on LSTM-CNN-CatBoost-GSSSA

**DOI:** 10.1371/journal.pone.0344678

**Published:** 2026-05-04

**Authors:** Xiaojuan Chen, Lufan Zhang, Xue Li, Funan Gao

**Affiliations:** 1 Changchun University of Science and Technology, Changchun, Jilin, China; 2 Changchun University of Science and Technology, Changchun, Jilin, China; 3 Changchun Institute of Technology, Changchun, Jilin, China; 4 State Grid Jilin Electric Power Co., Changchun, Jilin, China; The University of British Columbia, AUSTRALIA

## Abstract

Underground All-Dielectric Self-Supporting (ADSS) optical cables in urban areas are frequently compromised by external disturbances such as construction activities and human excavation, posing a serious threat to power grid security. Current single-algorithm approaches often struggle to adapt to the complex and variable urban environment, resulting in limited recognition accuracy. To address these challenges, this paper introduces LSTM-CNN-CatBoost-GSSSA, a hybrid recognition model that effectively captures the temporal, frequency, and spatial propagation characteristics of vibration signals. It integrates the sequential modeling capability of Long Short-Term Memory (LSTM) networks with the local feature extraction power of Convolutional Neural Networks (CNN). This layer generates adaptive weights through differentiable learning to realize the dynamic weighted fusion of time series and spatial-frequency domain features, thereby optimizing the feature selection mechanism. Moreover, an improved golden sine–enhanced Sparrow Search Algorithm (GSSSA) is employed to globally optimize CatBoost’s hyperparameters and adaptively adjust feature weights for enhanced recognition. Experimental results demonstrate that the proposed model achieves an accuracy of 97.6%, surpassing SVM, CNN, LSTM-CatBoost, CNN-CatBoost, LSTM-CNN-GBDT, and LSTM-CNN-CatBoost by 15%, 3.6%, 14.79%, 1.2%, 1.4%, and 0.49%, respectively. The model exhibits both high recognition performance and good computational efficiency, providing a feasible technical solution for real-time monitoring and intelligent diagnosis of external force damage to urban buried ADSS optical fiber cables. This advancement contributes to improving the operational safety and reliability of power communication networks.

## 1. Introduction

The rapid advancement of smart grid technology has led to a significant increase in the density of optical cables and fibers within urban power communication networks [1]. In particular, the All-Dielectric Self-Supporting (ADSS) optical cable is highly suitable for complex installation environments, including underground burial, and play a vital role in ensuring the safe and stable operation of the power grid. Nevertheless, the escalating operational risks pose considerable challenges to the safety of ADSS optical cables. A 2023 investigation by the State Grid Huangshan Power Supply Company identified human construction activities and natural disasters as the primary threats to line safety [2]. In densely populated urban areas, underground ADSS optical cables are highly susceptible to damage from frequent engineering activities such as excavation and mechanical vibration [3]. The interplay of extreme weather conditions and intensive human engineering operations results in a consistently high incidence of external damage to optical cables, thereby significantly jeopardizing the reliability and stability of power communication networks.

At present, the detection of external damage to ADSS optical cables primarily relies on traditional techniques such as manual inspection and video monitoring. However, these methods are plagued by low efficiency, delayed response, and high labor costs, limiting their suitability for real-time monitoring of extensive, long-haul optical cable networks. In contrast, Phase-Sensitive Optical Time Domain Reflectometry (φ-OTDR) detects vibrational parameters with high precision by analyzing Rayleigh backscatter variations in optical fibers, offering significantly enhanced stability and sensitivity for monitoring environmental changes [4–7]. Previous research has integrated φ-OTDR with various analytical approaches to enhance signal recognition accuracy. For instance, the combination of spectral subtraction and multi-feature extraction with Support Vector Machine (SVM) classification achieved recognition rates exceeding 90% for four types of vibration events [8]. Nonetheless, spectral subtraction is limited to initially suppressing a single noise type and struggles to effectively isolate valid external break signals amid the complex interference and construction vibrations typical of urban environments. Similarly, the application of Fourier decomposition alongside the Gradient Boosting Decision Tree (GBDT) algorithm yielded a recognition accuracy of 92.5% for four intrusion signal categories [9]; however, Fourier decomposition is confined to characterizing stationary frequency-domain features and fails to capture the instantaneous dynamic variations associated with external attack events such as mining and tapping. Further investigations utilizing public datasets by Xiaomin Cao et al. [10,11] demonstrated average recognition accuracies of 82.6% and 94.0% for SVM and Convolutional Neural Network (CNN) models, respectively, across six φ-OTDR event types. Despite this, SVM exhibits limitations in handling high-dimensional, complex data, with accuracy fluctuating under multiple interference conditions. CNN models, while more accurate, lack temporal feature capture capabilities, and their reliance on manual parameter tuning impedes real-time application efficiency. An in-depth analysis reveals that the bottleneck in current recognition performance primarily stems from three key factors: 1. Feature redundancy bottleneck: Under multi-source strong interference, such as construction noise and traffic, traditional spectral analysis and feature extraction methods struggle to effectively distinguish noise from damaged signals. This results in feature redundancy and a significant lack of discriminative power. 2. Limited adaptability of feature fusion: Most existing models employ a fixed multi-domain feature fusion strategy, which lacks the ability to adapt to dynamic environmental changes, thereby limiting the model’s generalization performance. 3. Global limitations in parameter optimization: In the context of a high-dimensional parameter space, relying on manual experience or a single optimization strategy is inefficient and prone to becoming trapped in local optima [12]. Consequently, achieving a balance between accuracy and efficiency is challenging. Currently, the integration of multi-domain information fusion with intelligent optimization algorithms represents a cutting-edge approach to enhancing the perceptual capabilities of complex systems. This approach has demonstrated significant advantages in image recognition, time series prediction, and multi-source data fusion prediction, including applications in hydrology and meteorology [13]. However, in the specific domain of φ-OTDR cable breakage monitoring, existing research tends to be fragmented and focused on isolated improvements. It fails to deeply integrate these approaches or establish a comprehensive framework that links adaptive feature fusion with global classifier optimization. Addressing this core issue is the primary objective of this paper. Reason: The revision improves clarity and readability by restructuring sentences and enhancing vocabulary. Technical terms are clarified, and the flow of ideas is made more logical and coherent. Punctuation and grammar errors are corrected, and the text is formatted for better comprehension without altering the original meaning.

To sum up, aiming at the recognition problem of external force damage sources vibration signals of ADSS underground optical cables in urban areas mentioned above, based on optical fiber sensing technology [14,15], this paper proposes a hybrid recognition model of LSTM-CNN-CatBoost-GSSSA. The first is to design the LSTM-CNN [16–19] three-domain feature gating fusion mechanism [20], dynamically adjust the weight distribution of temporal, frequency domain and spatial features, and enhance the model’s representation ability for complex signals; Second, the improved golden sine operator [21] is introduced in combination with the Sparrow search algorithm (GSSSA). Through the global exploration feature of the golden ratio and the overall optimization of sparrow swarm intelligence [22], the dynamic adjustment of multi-domain feature weights and the efficient optimization of Categorical Boosting (CatBoost) hyperparameters are achieved, and the precise recognition of external force damage sources vibration signals of underground ADSS optical cables in urban areas is completed.

## 2. LSTM-CNN-CatBoost-GSSSA recognition model

The central framework of the LSTM-CNN-CatBoost-GSSSA recognition model comprises two primary components: a three-domain feature fusion mechanism grounded in a gating strategy, and an optimization model that concurrently determines the multi-domain feature weights alongside the CatBoost hyperparameters. Initially, the LSTM-CNN architecture extracts features from the three distinct domains, which are subsequently integrated through the gating mechanism. The resulting fused features are then fed into the CatBoost model, whose parameters are optimized via the GSSSA algorithm, to generate the final recognition outcomes.

### 2.1. Three-domain feature extraction of LSTM-CNN

**Fig 1:**
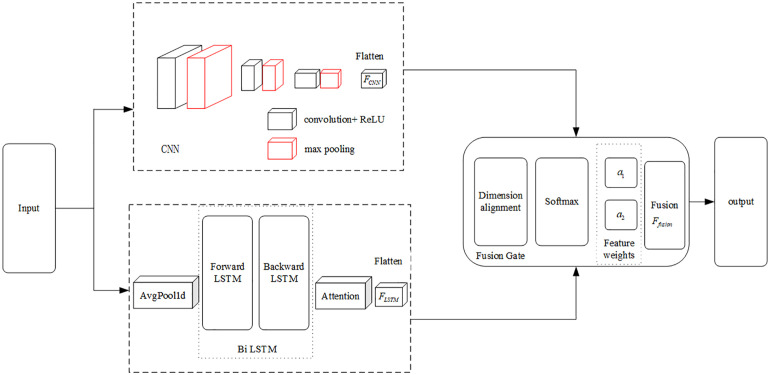
LSTM-CNN Feature extractor.

CNN are primarily utilized for feature extraction in both spatial and frequency domains, whereas Long Short-Term Memory (LSTM) networks excel in processing time series data [23–25]. This study employs a parallel architecture that integrates LSTM and CNN [16–19] to extract signal features through dual branches. Furthermore, a gated fusion mechanism is employed to enable the effective integration of features derived from temporal, frequency, and spatial domains. This architectural design maintains the distinctiveness of multi-domain features while simultaneously improving the comprehensive representation of information for subsequent vibration signal pattern recognition. The feature extraction model is illustrated in Fig 1, which visually depicts the parallel dual-branch feature extraction alongside the dynamic fusion mechanism.

The original vibration signal, acquired via φ-OTDR, serves as the input layer for shunt processing. The CNN branches utilize three convolutional neural networks to extract features from the spatial and frequency domains. The LSTM branch is structured as a Bi-directional Long Short-Term Memory (BiLSTM), which incorporates a downsampling layer that employs average pooling to reduce the sequence length, and integrates an attention mechanism [17] to enable the model to concentrate on significant temporal points. Following the computation of the weights, the temporal information features are derived. The formula for the attention mechanism is presented as follows:


$$e_{t}=\text{tanh}(W_{a}h_{t}+b_{a})$$ (1)

$$\alpha_{t}=\frac{exp\left(e_{t}\right)}{\overset{T}{\underset{k=1}{\sum }}exp\left(e_{k}\right)}$$ (2)

$$\text{context}=\sum_{t-1}^{T}\alpha_{t}h_{t}$$ (3)

Among them, input and parameters: $h_{t}$ is the hidden state of the first time step, that is, the output of LSTM. $W_{a}$ is the weight matrix. $b_{a}$ is the bias term. $e_{t}$ represents the attention energy of the t-th time step, and $e_{k}$ represents the attention energy of the k-th time step, reflecting the significance of this time step. The attention weight of $\alpha_{t}$ normalized by Softmax satisfies $\overset{T}{\underset{t=1}{\sum }}\alpha_{t}=1$. Output: context is the weighted summation of the context vector, integrating the information of all time steps.

In this study, a feature fusion layer is developed employing a gating unit. Spatial and frequency-domain features extracted via CNN and temporal features obtained from LSTM are integrated into a comprehensive feature representation through the gating mechanism. This fusion layer enables dynamic feature selection facilitated by differentiable gating. Specifically, the 128-dimensional output from the LSTM (the 256-dimensional output from a bidirectional LSTM) is combined with the 64-dimensional output from the CNN and subsequently compressed to 256 dimensions, rather than simply concatenating to 320 dimensions, via the gating mechanism. This approach preserves the local sensitivity characteristic of CNN and the temporal modeling capabilities of LSTM, while circumventing the constraints associated with manually designed fusion rules by leveraging a soft attention mechanism. The proposed method is particularly suited for processing multi-domain heterogeneous features.

The original calculation formula for gated convolution is [20]:


$$G_{y,x}=\sum \sum W_{g}\cdot F_{I}$$ (4)

$$F_{y,x}=\sum \sum W_{f}\cdot F_{I}$$ (5)

$$O_{y,x}=\phi \left(F_{y,x}\right)⊙\sigma \left(G_{y,x}\right)$$ (6)

Among them, the input feature map is represented as $F_{I}$, $W_{g}$ and $W_{f}$are two convolution operations on $F_{I}$, and is the weight matrix of the gated convolution and feature convolution, respectively. $G_{y,x}$ is the gated value, $F_{y,x}$ is the result of feature transformation. With the activation functions being ϕ and σ. The results of the two convolution operations are processed respectively using the activation function ϕ and the activation function σ, and finally the output $O_{y,x}$ is generated by multiplying the corresponding elements.

Building upon the aforementioned gating mechanism and considering the heterogeneity of LSTM and CNN features addressed in this study, the original gating formula has been modified and enhanced. Initially, the features extracted from the two branches are dimensionally aligned and concatenated. Subsequently, feature weights are learned via the gating layer, enabling dynamic fusion. The detailed derivation process of the gated fusion formula proposed herein is as follows:

(1) Calculate the gating value

In order to achieve gated fusion of LSTM with 256 output dimensions and CNN with 64 output dimensions, align and concatenate feature vectors after dimension unification, and then calculate the gated value G:


$$G=W\cdot \left[\begin{array}{c} @c@F_{CNN}\\ F_{LSTM} \end{array}\right]+b$$ (7)

Among them, $F_{CNN}$ and $F_{LSTM}$ are the processed features of the corresponding model, $\left[\begin{array}{c} @c@F_{CNN}\\ F_{LSTM} \end{array}\right]$ is the concatenated feature vector, W is the weight matrix, and b is the bias term, which is linearly transformed to learn the gating information.

(2) Compute the feature weights

Softmax function can transform the numerical value in G into a probability value between 0 and 1, $F_{CNN}$ and $F_{LSTM}$ the sum of all probability values is 1, which represents the weight proportion of $F_{CNN}$ and $F_{LSTM}$ in the final fusion feature. The Softmax function is applied to the gated value G to obtain the feature weights a as follows:


a=Softmax(G) (8)

(3) Dynamic feature fusion

$$F_{fusion}=a_{\text{1}}⊙F_{CNN}+a_{\text{2}}⊙F_{LSTM}$$ (9)

Among them, $a_{\text{1}}$ and $a_{\text{2}}$are two elements in the feature weight a, corresponding to the weights of $F_{CNN}$ and $F_{LSTM}$, respectively. The corresponding elements are multiplied to obtain two features with weight adjustment, and the final fusion feature $F_{fusion}$ is obtained by adding them. In this way, the weights of the two features are dynamically adjusted according to the gating value, so that the multi-domain information can be flexibly fused.

The innovation of this gated fusion layer is demonstrated in the following three aspects:

1. Dynamic Adaptive Weight Allocation: Unlike traditional fixed-weight fusion, this method dynamically adjusts the weights of the two branches based on the characteristics of each input sample through a gating mechanism. This allows the model to flexibly respond to different types of events. For transient events, it emphasizes spatio-temporal features, while for continuous vibration events, it focuses more on temporal characteristics.
2. End-to-end differentiable training: The entire gated fusion layer, along with the preceding feature extraction network and the subsequent classifier, constitutes an end-to-end trainable framework. This framework optimizes the gating parameters through backpropagation, enabling it to learn the optimal fusion strategy directly from the data without human intervention.
3. Feature Selection and Noise Reduction: The gating mechanism functions as a feature selector, enhancing important features and suppressing redundant ones through weight allocation, thereby improving the discriminative power of the fused features.

### 2.2. GSSSA optimizes the CatBoost process

**Fig 2:**
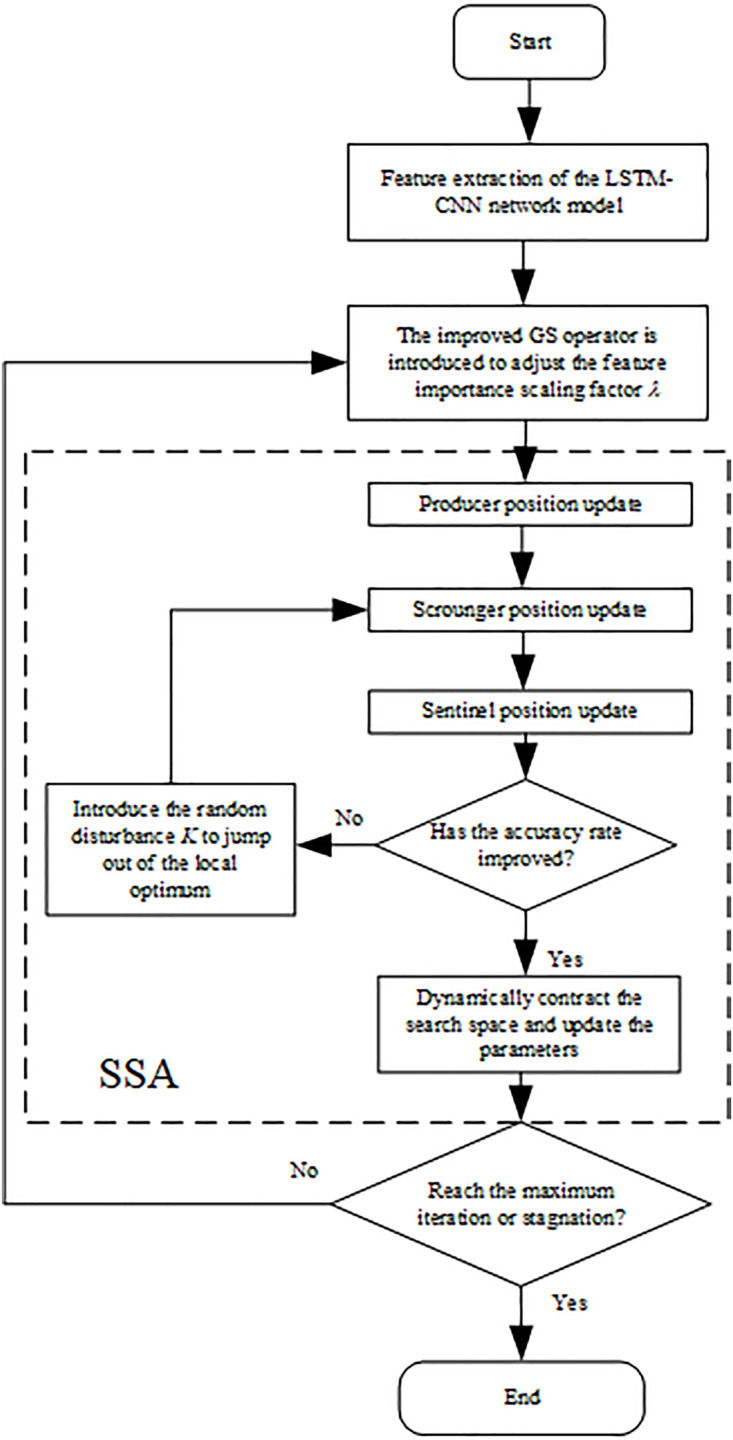
Flowchart of the GSSSA optimization framework.

Aiming at the multi-domain characteristics and recognition problem of the vibration signal of φ-OTDR optical cables, this paper proposes a sparrow search Optimization Framework that introduces the improved golden sine operator (GSSSA Optimization Framework). Through the joint optimization of dynamic feature weight allocation and hyperparameters, as shown in Fig 2, this section introduces the weight allocation and parameter optimization.

The core of the optimization framework in Fig 2 is: introducing the improved golden sine operator, based on the golden ratio and the characteristics of the sine function, adaptively optimizing the multi-domain feature weights, and constructing a dynamic weight adjustment layer; Construct a sparrow search hyperparameter optimization layer to optimize the CatBoost hyperparameters through the swarm intelligence behavior of sparrows and avoid local optima.

The Golden Sine Operator (GS) dynamically adjusts the search step size through the golden ratio $\tau =(\sqrt{\text{5}}-1)/2$ and combines the nonlinear mapping characteristics of the sine function to achieve a balance between global exploration and local development of feature weights [21]. Its position update formula is:


$$\overset{t+1}{\underset{i}{w}}=\overset{t}{\underset{i}{w}}\cdot sin\left(\tau \cdot \pi \cdot r_{\text{1}}\right)+\left|\begin{array}{c} \tau \cdot x_{best}-r_{\text{2}}\cdot \overset{t}{\underset{i}{w}} \end{array}\right|$$ (10)

Among them, $r_{\text{1}}$ and $r_{\text{2}}$ are random numbers, and $x_{best}$ is the current optimal weight vector. $\overset{t}{\underset{i}{w}}$ is the weight of the i-th feature in the t-th iteration. Through the position update formula of the golden sine operator, the dynamic adjustment of the weight of each feature is realized.

For a high-dimensional fused feature space, directly applying the standard golden sine operator to update each feature dimension independently results in high computational complexity. Therefore, this paper introduces a global feature importance scaling factor, *λ*, to address this issue. The optimal value of *λ* was efficiently determined using the golden section method within the interval [0.1, 2.0]. The optimization process employed a tolerance (tol) of 0.01 and a maximum of 20 evaluations to prevent overfitting. Given the computational demands of the golden sine formula and the challenge of rapid calculation in the high-dimensional fused feature space, this study uses the golden section method to optimize a unified feature importance scaling factor rather than adjusting each weight individually. The weight mapping $w_{i}$ of the sine function is improved to:


$$w_{i}=\left|sin\left(\lambda \cdot \frac{\pi }{\text{2}}\cdot \frac{\text{1}}{max\left(w\right)}\right)\right|$$ (11)

The weight is controlled by a single parameter λ to reduce the computational complexity of the high-dimensional feature space: The nonlinear characteristics of the sine function highlight the important features, and the weights are normalized by max(w) to avoid numerical overflow. This design reduces the computational complexity from 𝑂(𝑑) to 𝑂(1). The parameter optimization of 𝑂(1) level is combined with the vectorization operation of 𝑂(𝑑) to enhance optimization efficiency. The value of 𝜆 is determined by minimizing the negative cross-validation accuracy (i.e., maximizing the accuracy).

The Sparrow Search Algorithm (SSA), as a population intelligence optimization algorithm, is based on the simulation of foraging and anti-predation behaviors of sparrow populations. The core idea is to achieve global optimization through Producer, Scrounger, and Sentinel [22]. The mathematical modeling is as follows [12]:

(1) Introduce Producer. The Producer is obtained first during the search process and can be searched more extensively. The position update formula is:

$$\overset{t+1}{\underset{i,j}{X}}=\left\{ \begin{array}{cc} @cc@X_{i,j}\cdot exp\left(-\frac{i}{b\cdot ite{\text{m}}_{max}}\right) & R_{\text{2}}<ST\\ X_{i,j}+Q\cdot L & R_{\text{2}}\geq ST \end{array}\right.$$ (12)

Among them, $X_{i,j}$ represents the information of the i-th individual in the j-th dimension, t is the current iteration, $ite{\text{m}}_{max}$ is the set maximum iteration, and b is a constant whose value is within (0，1]. Q is a constant that follows a normal distribution, and L is a matrix where all elements are 1 and the columns are the same as the initialization matrix. $R_{\text{2}}$ and ST are respectively the alarm value and the safety value, which are used to determine whether the position needs to be changed.

(2) Introduce Scrounger. The Scrounger follows the Producer and compets with it to obtain the Producer’s achievements. The position update formula is:

$$\overset{t+1}{\underset{i,j}{X}}=\left\{ \begin{array}{cc} @cc@Q\cdot exp\left(\frac{X_{\text{w}}-\overset{t}{\underset{i,j}{X}}}{i^{\text{2}}}\right) & i>n/2\\ \overset{t+1}{\underset{\text{P}}{X}}+|\overset{t}{\underset{i,j}{X}}-\overset{t+1}{\underset{P}{X}}|\cdot A^{+}\cdot L & i\leq n/2 \end{array}\right.$$ (13)

Among them, $\overset{t+1}{\underset{\text{P}}{X}}$ is the current optimal position information of the Producer, A satisfies $A^{+}=A^{T}{\left(AA^{T}\right)}^{-1}$, and is a 1×d matrix with elements randomly 1 or −1, and $X_{\text{w}}$ is the global worst position information.

(3) Introduce Sentinel. Sentinel refers to the individual parts of the sparrow that are at the edge and vulnerable to attacks, causing alerting. When they are in danger, they gather together. The position is updated to:

$$\overset{t+1}{\underset{i,j}{X}}=\left\{ \begin{array}{cc} @cc@\overset{t}{\underset{\text{b}}{X}}+\beta \cdot |\overset{t}{\underset{i,j}{X}}-\overset{t}{\underset{\text{b}}{X}}| & f_{i}>f_{\text{b}}\\ \overset{t}{\underset{i,j}{X}}+K\cdot \left(\frac{\left|\overset{t}{\underset{i,j}{X}}-\overset{t}{\underset{\text{w}}{X}}\right|}{\left(f_{i}-f_{\text{w}}\right)+\varepsilon }\right) & f_{i}=f_{\text{b}} \end{array}\right.$$ (14)

Among them, $\overset{t}{\underset{\text{b}}{X}}$ is the global optimal position information, $f_{\text{b}}$ is the individual optimal fitness value, and β and ε are constants. At the end of the training, a stagnation detection mechanism is set up. If there is no improvement in the optimization objective for two consecutive times, a random perturbation is applied to the feature weights, that is, the perturbation intensity K is implicitly simulated through a uniformly distributed random number within a certain range.

Following the processes of feature extraction and model optimization, the proposed LSTM-CNN-CatBoost-GSSSA model is employed for precise pattern recognition. This model is predicated on the LSTM-CNN feature extraction network and utilizes CatBoost [26] as the recognition algorithm. CatBoost is a boosting-type ensemble learning algorithm that builds upon the enhanced Gradient Boosting Decision Tree (GBDT) [12]. It adeptly handles categorical features through ordered target statistics, thereby mitigating the risks of information loss and overfitting. Additionally, the model employs an ordered lifting technique to rectify gradient estimation bias, while a symmetric binary tree structure is integrated to bolster the model’s generalization capabilities [14,27]. To facilitate the accurate classification of vibration signals originating from external sources, multiple decision tree models undergo continuous optimization via iterative training, ensuring that the output aligns closely with real-world conditions. Utilizing the φ-OTDR vibration signal dataset, this study develops a recognition model for external force damage sources affecting optical cables and introduces the GSSSA optimization method for comprehensive tuning of the CatBoost model.

## 3. Experimental simulation

### 3.1. Experiment preparation

This study utilizes the φ-OTDR open-source dataset referenced in [10] (GitHub: https://github.com/BJTUSensor/Phi-OTDR_dataset_and_codes). The φ-OTDR experimental data acquisition covered six distinct events, labeled 0–5: background noise, digging, knocking, watering, shaking, and walking. Among these, digging and knocking were identified as critical external breakage incidents. Data collection was conducted under the following conditions:

(1) Background noise: Recorded in a controlled laboratory environment.
(2) Digging: A 10-meter fiber section buried in a 15 cm deep sandbox at the end of a 5/10 km sensing fiber was disturbed via simulated excavation.
(3) Knocking: A 10-meter fiber coil on an anti-vibration platform was struck with a hammer.
(4) Watering: Water was sprayed uniformly onto the sensing fiber from a height of 30 cm.
(5) Shaking: The fiber was fixed to a fence and rhythmically shaken at various points.
(6) Walking: Personnel walked or ran within a 20-meter radius at the distal end of the fiber.

The resulting dataset comprises 15,419 samples, each representing a 10,000 (temporal) × 12 (spatial) matrix. Samples were distributed as: background noise (2,946), digging (2,512), knocking (2,530), watering (2,253), shaking (2,728), and walking (2,450). An 8:2 split yielded 12,335 samples for training and 3,084 for testing, mitigating class imbalance and enabling an objective evaluation of model generalization.

**Fig 3:**
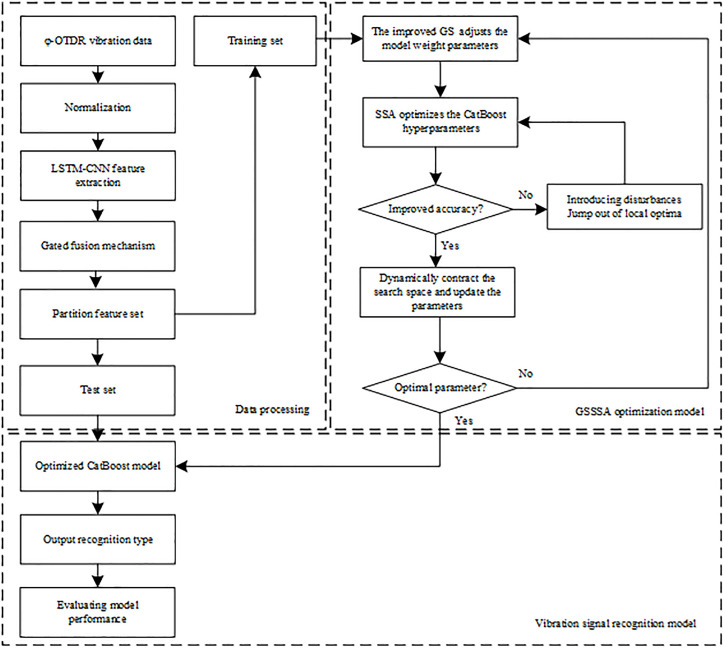
Block diagram for identifying external force damage sources vibration signals.

This study integrates deep learning and ensemble learning methodologies, specifically employing a LSTM-CNN feature gating mechanism, alongside CatBoost for recognition, and utilizes the GSSSA for optimization, to investigate pattern recognition within the φ-OTDR system. The procedural steps outlined in the block diagram presented in Fig 3 are as follows:

Step 1: Data processing. The vibration signals obtained from the φ-OTDR are normalized. A deep learning model, specifically an LSTM-CNN with a gated fusion mechanism, is designed to extract feature vectors. The dataset is partitioned into a training set and a test set in an 8:2 ratio.

Step 2: An improved golden sine operator is introduced to dynamically modify the feature weights.

Step 3: The Sparrow Search Algorithm is employed to optimize the hyperparameters of the CatBoost model.

Step 4: Determine whether the accuracy rate of the optimization target has improved. If an improvement is observed, the search space is dynamically adjusted, and parameters are updated. Conversely, if no improvement is detected, perturbations are introduced to escape local optima, feature weights are adjusted through backpropagation, and Step 2 is reiterated.

Step 5: Determine whether the maximum iteration or stagnation has been reached. If so, the optimization process is concluded, and the optimal parameters for the CatBoost model are outputted. If the termination criteria are not satisfied, Steps 2–5 are repeated.

Step 6: The optical cable vibration signal classification model is applied to the test set, producing classification results and conducting a performance evaluation.

The experiment was conducted utilizing a high-performance computing platform. The detailed specifications of the software and hardware configurations are presented in Table 1 below:

**Table 1:** Experimental software and hardware configuration.

Hardware configuration	Software environment
NVIDIA GeForce RTX 3060 Laptop GPU	Python 3.12.0
Intel i7-12700H CPU	PyTorch 2.3.0 + cu118
16GB (DDR4/5)	CUDA 11.8

The aforementioned configuration facilitates the effective training of deep learning models and meets the parallel computing demands of intricate algorithms, thereby offering hardware assurances for the dependability of experimental outcomes.

### 3.2. Feature extraction network

The CNN component is designed to extract features from the spatial-frequency domain through a series of three convolutional layers. The first convolutional layer employs a large kernel size of (200, 3), spanning 200 temporal sampling points and integrating all 12 spatial channels to initially capture the joint spatiotemporal patterns of vibration signals. The kernel sizes of the subsequent two convolutional layers are progressively reduced to (20, 2) and (3, 1), focusing on extracting finer local frequency-domain features. The number of channels in the network increases sequentially from 16 to 32 and then to 64, gradually enhancing the feature representation capability. Each convolutional layer is followed by BatchNorm2d, ReLU activation, MaxPool2d, and Dropout with a probability of 0.2. BatchNorm2d stabilizes the training process and accelerates convergence; the ReLU function is chosen for its efficiency in mitigating vanishing gradients, and Dropout is applied to prevent model overfitting. This component is designed purely as a feature extractor without a fully connected layer at the end and ultimately outputs a 64-dimensional feature vector for subsequent fusion. The training of this model employs the cross-entropy loss function (nn.CrossEntropyLoss) with a batch size of 32. The Adam optimization algorithm is utilized, with an initial learning rate set at 0.001. A learning rate scheduler is incorporated, which reduces the learning rate by half every two epochs if there is no improvement in the validation loss. The initial training duration is established at 50 epochs, with an early stopping criterion implemented; if the validation loss does not show improvement after a specified patience period of 5 epochs, training will be halted prematurely.

The LSTM component employs a two-layer bidirectional LSTM architecture combined with an attention mechanism to effectively extract temporal features. The original signal sequence length can be as long as 10,000. To reduce computational complexity, a one-dimensional average pooling layer with a downsampling factor of 10 (AvgPool1d) is first applied, reducing the sequence length to 1,000. The processed sequence is then fed into the two-layer bidirectional LSTM network, which has an input feature dimension of 12 and a hidden state dimension of 128. The bidirectional structure enables the integration of forward and backward information at each time step, resulting in a final output dimension of 256 per time step. To enhance the model’s ability to focus on critical time steps, an attention mechanism is applied after the LSTM output. This mechanism consists of a linear layer (mapping the dimension from 256 to 64), a Tanh activation, a linear layer (mapping the dimension from 64 to 1), and Softmax normalization, ultimately producing a 256-dimensional weighted temporal feature vector.

At the final stage of the model, a multi-domain feature gating fusion mechanism is introduced. This mechanism combines the spatial and frequency-domain features extracted by the CNN with the temporal features extracted by the LSTM network. By integrating these features through the gating fusion mechanism, the model enhances its capability to perform classification tasks by leveraging spatio-temporal information effectively.

To visually assess the effectiveness of various feature extraction modules, this paper employs a two-stage dimensionality reduction strategy to visualize high-dimensional features. First, all original vibration signals are standardized using Z-score normalization before being input into the network, eliminating dimensional effects and enhancing training stability. When the feature dimension exceeds 50, PCA is initially applied to reduce the feature space to 50 dimensions, thereby removing redundant information. Subsequently, t-SNE mapping [28] is employed to project the features into a two-dimensional space. The t-SNE parameters are set as follows: perplexity of 30, learning rate of 200, and 1000 iterations. The underlying mathematical principle is described as follows:


$$z_{\text{tsne}}=arg\;minKL\left(P\right|\left|Q\right)\text{\textbackslash{}hspace\{0.33em}\;\}$$ (15)

Among them, P represents the similarity distribution in the high-dimensional space, Q represents the similarity distribution in the low-dimensional space, and KL divergence measures the difference between the two.

**Fig 4:**
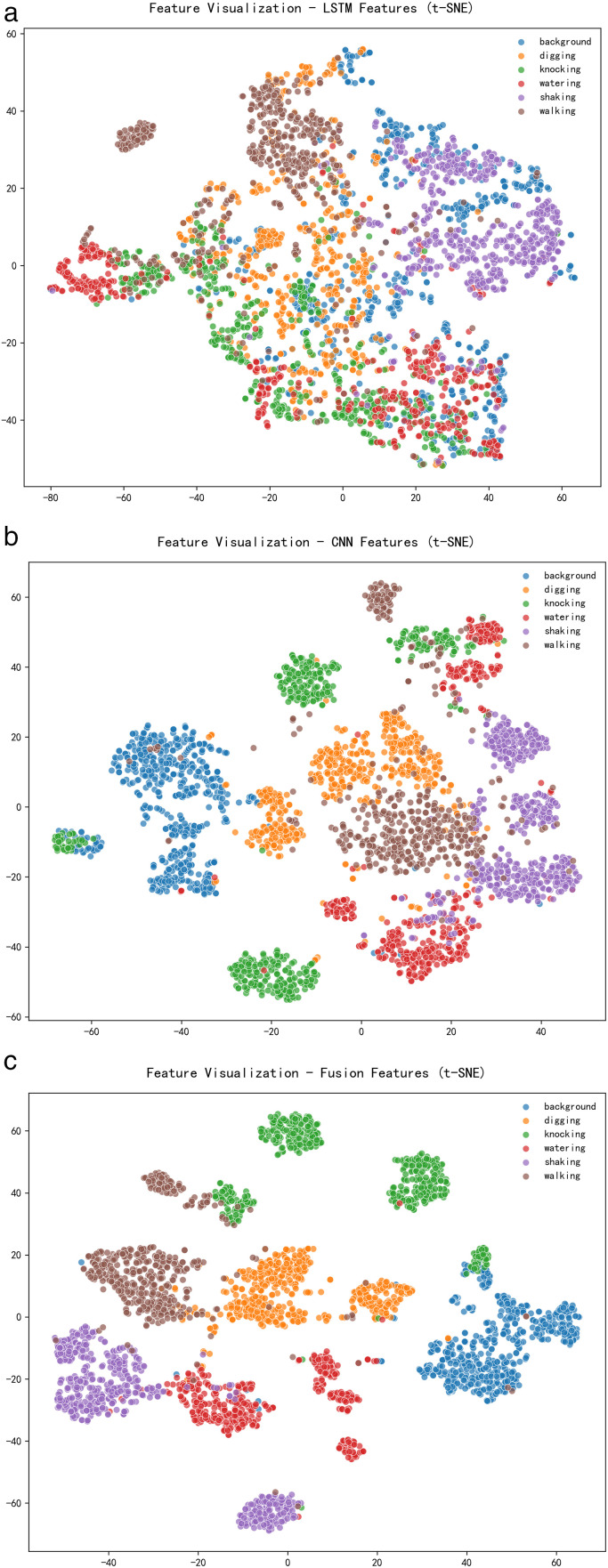
Distribution map of t-SNE characteristics. (a) LSTM features (b) CNN features (c) LSTM-CNN fusion features.

The two-dimensional t-SNE visualizations of features extracted by LSTM, CNN, and the proposed LSTM-CNN fusion layer in this study are presented in Fig 4. Based on image analysis, the temporal continuity reflected in the LSTM feature t-SNE distribution demonstrates an effective ability to capture time-series information within time-series data, enabling similar temporal patterns to cluster in low-dimensional space. However, the reliance on long-term dependencies inherent to LSTM results in blurred category boundaries, complicating the differentiation of specific event distinctions. Conversely, the t-SNE distribution of CNN features exhibits strong clustering in the frequency domain, effectively extracting local features from time-series data. This increases inter-class distances while reducing intra-class distances, thereby forming relatively distinct clusters for different classes. Nevertheless, the small distance between mining and walking classes leads to ambiguous boundaries, which may hinder the precise identification of external force damage sources. In contrast, the t-SNE visualization of the LSTM-CNN fusion features presents a clearer decision boundary. It not only enhances inter-class separation, addressing the issue of blurred boundaries between external disruption events and other events, but also strengthens the model’s capacity to distinguish various event types.

### 3.3. Analysis of the recognition algorithm results

On the φ-OTDR abnormal event dataset, the classical deep learning CNN end-to-end recognition model was compared with the LSTM-CNN end-to-end recognition model. The recognition accuracy using only the CNN network reached 94.0%, whereas the recognition accuracy of the LSTM-CNN network, which incorporates time-series information, improved to 95.1%. Experimental results demonstrate that introducing temporal information significantly enhances the recognition performance for vibration signals.

From the preceding analysis, it is evident that the recognition performance of CatBoost is influenced by random input weights and hyperparameter settings. To address this issue, the GSSSA optimization algorithm was employed to optimize CatBoost, integrating a feature extraction network to construct the hybrid recognition model LSTM-CNN-CatBoost-GSSSA. This approach dynamically adjusts feature weights through an improved golden sine operator, thereby amplifying the contribution of key features to classification decisions. Subsequently, the global optimization mechanism of the sparrow search algorithm is employed to systematically optimize the core hyperparameters of CatBoost, including the learning rate (0.01 to 0.2), tree depth (4–10), and number of iterations (100–1000). During the optimization process, the sparrow population size is set to 30, the maximum number of iterations is 50, and the dynamic golden sine coefficient λ is linearly increased from 0.1 to 1.0 to balance exploration and exploitation. Through this dual optimization mechanism for both feature weights and hyperparameters, the recognition accuracy of the model in complex vibration signal pattern recognition was effectively improved.

To quantitatively verify the contribution of each core component proposed in this paper, an ablation experiment was designed: the Adam optimizer (β₁ = 0.9, β₂ = 0.999, weight decay coefficient = 1e-5) was used, combined with a scheduling strategy and an early stopping mechanism to prevent overfitting. After multiple rounds of iterative training, the LSTM-CNN-CatBoost-GSSSA model achieved an average accuracy of 97.60% on the test set comprising six types of events, validating the efficacy of the proposed optimization strategy. Based on the recognition model combining deep learning and ensemble learning, this study conducted a comparative analysis of the final models, including LSTM-CatBoost, CNN-CatBoost, LSTM-CNN-CatBoost, and LSTM-CNN-CatBoost-GSSSA, as presented in Table 2.

**Table 2:** Comparison of classification performance indicators of recognition models.

Recognition model	Accuracy	Precision	Recall	F1
LSTM-CatBoost	82.81%	82.53%	82.54%	82.53%
CNN-CatBoost	96.40%	96.41%	96.34%	96.37%
LSTM-CNN-CatBoost	97.11%	97.05%	97.06%	97.05%
LSTM-CNN-CatBoost-GSSSA	97.6%	97.5%	97.67%	97.33%

The evaluation criteria such as accuracy rate, precision rate, recall rate and F1 score used for model performance assessment, as well as formula verification [15]:


$$Accuracy=\frac{TP+TN}{TP+TN+FP+FN}$$ (16)

$$Precsion=\frac{TP}{TP+FP}$$ (17)

$$Recall=\frac{TP}{TP+FN}$$ (18)

$$F1=\frac{2Precsion\times Recall}{Precsion+Recall}$$ (19)

Among them, TP is a true positive, FP is a false positive, FN is a false negative, and TN is a true negative. As shown in Table 2, the model’s performance demonstrates a clear stepwise improvement trajectory. The detailed analysis is as follows:

1. Establishment of a single-domain feature baseline: Using CatBoost as the unified classifier, the accuracy of the LSTM-CatBoost model, which utilizes only temporal features, is 82.81%. In contrast, the CNN-CatBoost model, which uses only spatial-frequency domain features, achieves an accuracy of 96.40%. The significant gap between the two (13.59 percentage points) indicates that the discriminative power of spatial-frequency domain features is substantially better than that of time series features. However, a single feature domain cannot fully characterize complex vibration signal patterns.
2. Verification of the gated fusion mechanism: Building on the CNN-CatBoost model, an LSTM branch is introduced, and a differentiable gated fusion layer is embedded to construct the LSTM-CNN-CatBoost model. The accuracy increases to 97.11%, and the F1 score correspondingly improves by 0.68 percentage points to 97.05%. This result demonstrates that the gated fusion mechanism effectively integrates dual-domain information, compensates for the limitations of single features, and significantly enhances the model’s comprehensive representation capability.
3. Performance gain from the GSSSA optimization strategy: The GSSSA algorithm is further introduced to co-optimize feature weights and hyperparameters, forming the final LSTM-CNN-CatBoost-GSSSA model. Precision improves further to 97.60%, and recall increases by 0.61 percentage points to 97.67%. This clearly shows that GSSSA effectively strengthens the model’s recognition ability for key samples (especially external disaster events) and optimizes the decision boundary by adaptively adjusting feature importance and classifier parameters. Ablation experiments successively verify the independent and incremental contributions of dual-domain feature fusion and intelligent optimization algorithms to the model’s performance, systematically confirming the rationality and effectiveness of the proposed hybrid architecture and optimization strategy design.

**Fig 5:**
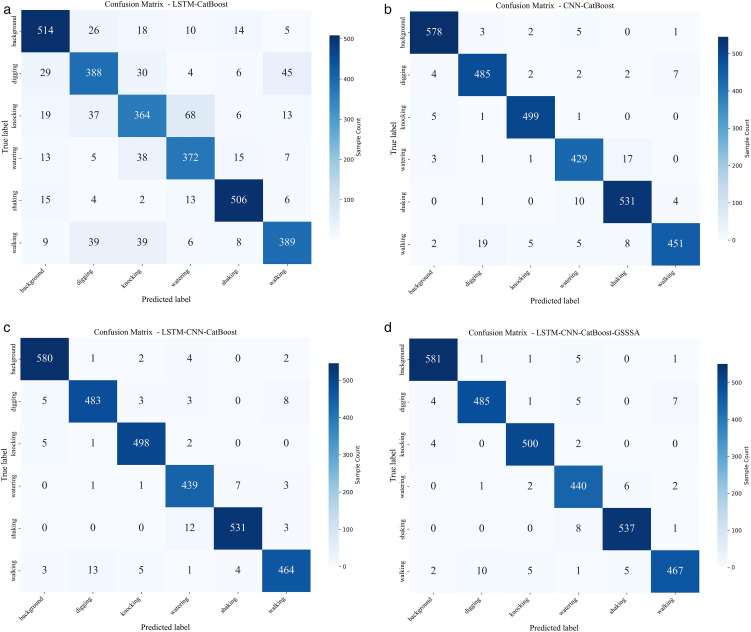
Confusion matrix diagram. (a) LSTM-CatBoost (b) CNN-CatBoost (c) LSTM-CNN-CatBoost (d) LSTM-CNN-CatBoost-GSSSA.

The confusion matrix is shown in Fig 5, where each cell represents the number of sample classifications. To clearly compare the performance of each model in identifying key external events, the evolution of the misclassification rate is quantified by analyzing the row values corresponding to two key event types—digging and knocking—based on the confusion matrices of the four models presented in Fig 5. From LSTM-CatBoost to CNN-CatBoost, the misclassification rate for key events decreases sharply (digging from 22.7% to 3.4%, and knocking from 28.2% to 1.4%), and the error pattern shifts from “multi-class dispersion” to which confirms the core discriminative power of spatial-frequency domain features. The LSTM-CNN-CatBoost model further concentrates misclassifications into a few easily confused categories (such as background and walking) through gated fusion while maintaining high accuracy, reflecting the optimization effect of multi-domain information complementarity on the decision boundary. Finally, the LSTM-CNN-CatBoost-GSSSA model increases the number of correctly classified percussion samples to 500 and reduces the misclassification of the two key event types to a very low level. This demonstrates that the collaborative optimization strategy of GSSSA effectively filters feature noise and sharpens the classification boundary, thereby providing a solid foundation for achieving highly reliable crack monitoring.

The evaluation results of six types of vibration events, namely background, digging, knocking, watering, shaking and walking (labels corresponding to 0–5), in the above four models are shown in Tables 3–6:

**Table 3:** Evaluation results of LSTM-CatBoost.

Label	Precision	Recall	F1
0	85.81%	87.27%	86.53%
1	79.02%	77.29%	78.15%
2	75.15%	75.89%	75.52%
3	80.39%	82.71%	81.53%
4	91.17%	92.67%	91.92%
5	83.84%	79.39%	81.55%

**Table 4:** Evaluation results of CNN-CatBoost.

Label	Precision	Recall	F1
0	97.64%	98.13%	97.88%
1	95.10%	96.61%	95.85%
2	98.04%	98.62%	98.33%
3	94.91%	95.12%	95.02%
4	95.16%	97.25%	96.20%
5	97.41%	92.04%	94.65%

**Table 5:** Evaluation results of LSTM-CNN-CatBoost.

Label	Precision	Recall	F1
0	97.81%	98.47%	98.14%
1	96.79%	96.22%	96.50%
2	97.84%	98.42%	98.13%
3	95.23%	97.34%	96.27%
4	97.97%	97.25%	97.61%
5	96.67%	94.69%	95.67%

**Table 6:** Evaluation results of LSTM-CNN-CatBoost-GSSSA.

Label	Precision	Recall	F1
0	98.31%	99.64%	98.47%
1	97.59%	96.61%	97.10%
2	98.23%	98.81%	98.52%
3	95.44%	97.56%	96.49%
4	97.99%	98.35%	98.17%
5	97.70%	95.31%	96.49%

The limitations of the single-feature model and the correlation analysis of event characteristics are clearly demonstrated by comparing the results of LSTM-CatBoost (Table 3) and CNN-CatBoost (Table 4). For two types of key external breaking events—digging (Label 1) and knocking (Label 2)—the core discriminative information in the vibration signals is primarily reflected in the spatial propagation patterns and the energy concentration within specific frequency bands. Therefore, the CNN model, which excels at extracting local spatial and frequency-domain features, performs significantly better than the LSTM model, which relies on long-term temporal dependencies for these events. The F1 score difference between the two models is 17.70 and 22.81 percentage points, respectively. Conversely, for background (Label 0), which is spatially uniform but may exhibit transient anomalous perturbations in the temporal sequence, the LSTM’s ability to capture long-range context makes it slightly better than CNN in terms of recall. This confirms the fundamental limitation that a single feature extractor struggles to fully meet the discrimination requirements posed by the heterogeneity of multi-class events.

According to the analysis in Table 5, the LSTM-CNN-CatBoost model achieves comprehensive performance improvement and balance through the gated fusion mechanism. Specifically, for the walking event characterized by weak and unstable vibration patterns (Label 5), the pure CNN model cannot effectively capture its intermittent temporal patterns, resulting in a low recall of 92.04%. By incorporating the temporal modeling capability of the LSTM branch, the fusion model improves recall by 2.65 percentage points, reaching 94.69%. Meanwhile, for mining and tapping events with prominent spatial features, the fusion model further refines the decision boundary by leveraging temporal context alongside the high accuracy of the CNN, maintaining F1 scores at elevated levels of 96.50% and 98.13%, respectively. This demonstrates that the gated fusion layer can dynamically adjust the contribution weights of spatio-temporal and temporal features based on the intrinsic characteristics of events, thereby achieving complementarity across different event discrimination blind spots.

As shown in Table 6, the LSTM-CNN-CatBoost-GSSSA model achieves a significant performance breakthrough compared to the fusion model, particularly in detecting two types of external attacks with critical security implications: digging and knocking. The precision rates reach 97.59% and 98.23%, while the recall rates are 96.61% and 98.81%, respectively. Compared to the fusion model, the F1 scores improve by 0.60 and 0.39 percentage points. This enhancement is primarily attributed to the dual optimization effect of the GSSSA algorithm. First, the improved golden sine operator dynamically adjusts feature weights to strengthen spatial-frequency domain features that are highly discriminative for external damage events, while suppressing interference from non-key features. Second, the sparrow search algorithm globally optimizes CatBoost hyperparameters, enabling the classifier to more accurately fit the feature distribution after weight screening, thereby forming a sharper decision boundary. These results confirm that the proposed optimization strategy not only improves overall performance but also enhances the model’s sensitivity and reliability in identifying critical security threat events.

According to the evaluation results presented in Tables 3, 4, 5, and 6, the performance evolution path and its underlying mechanisms—from the single-feature model to the multi-domain fusion model, and subsequently to the intelligent optimization model—are elucidated. Additionally, the contributions of different model components to specific event types are clearly identified.

**Fig 6:**
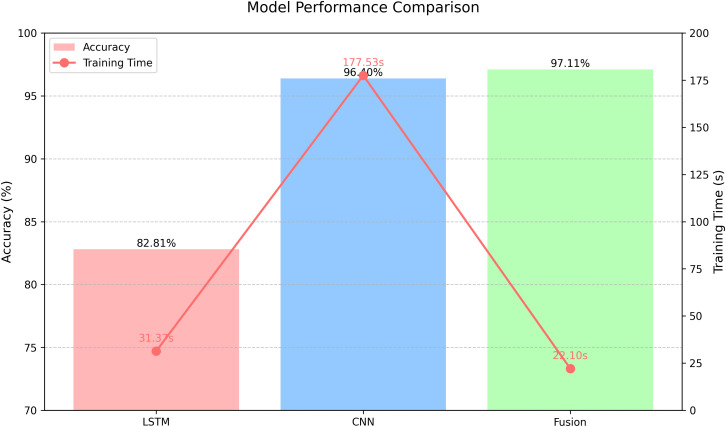
Bar line chart of accuracy-training time.

The efficiency advantage of the LSTM-CNN-CatBoost hybrid model in this paper is reflected in the gated fusion mechanism. If the dimensions are simple concatenation, the 256-dimensional features of LSTM are concatenated with the dimensions of CNN to form 512-dimensional features directly input into the subsequent module, and the subsequent processing complexity is O(512·M), M is the parameters of the subsequent layer. After gated fusion, the feature dimension is compressed to 256, and the complexity of subsequent processing is reduced to O(256·M). Dynamic weight allocation is used to avoid redundant features from participating in the calculation and improve the efficiency of the model. The columnar line chart of its comparison with the single LSTM-CatBoost model and CNN-CatBoost model accuracy-training time is shown in Fig 6.

According to Fig 6, the training time of LSTM-CNN gated fusion feature combined with CatBoost is only 22.10s, which is far lower than 177.53s of single CNN-CatBoost, and the optimal number of iterations obtained by the experiment is only 246. It is lower than 405 times of LSTM-CatBoost and 750 times of CNN-CatBoost, reflecting the efficiency advantage of the proposed model. The GSSSA optimization increment is controllable, the original golden sine operator is updated element by element, and the complexity is O(d·S·T), where d is the parameter dimension, S is the population size, and T is the number of iterations. After improvement, it is controlled by a single parameter λ, and the complexity is reduced to O(S·T). In the scenario with the same data set and task, GSSSA only needs to optimize the parameters once, and the optimal parameters obtained can be directly used for subsequent model training and inference without repeated calculation. That is, in practical applications, the optimization overhead of GSSSA is only generated when the model is deployed for the first time, and there is no additional complexity in the subsequent large-scale data inference stage, which will not affect the real-time performance of the model.

**Fig 7:**
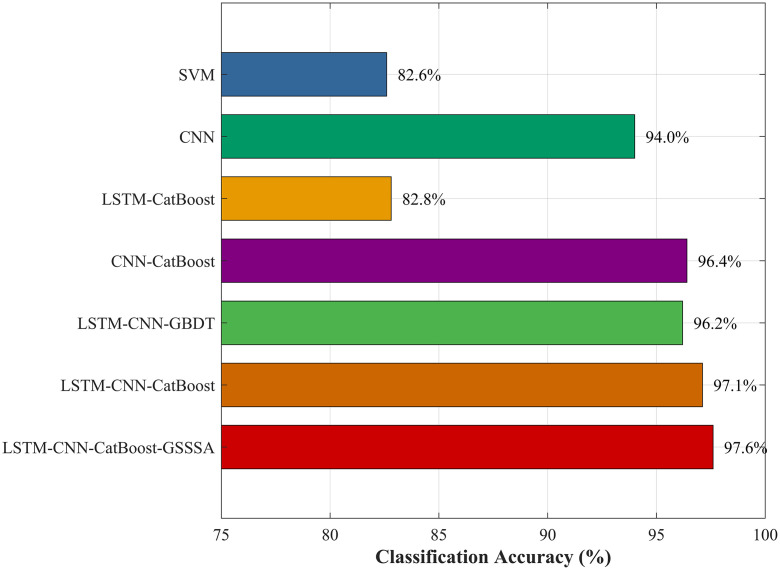
Comparison chart of the recognition accuracy of seven types of algorithms.

The recognition methods SVM, CNN, and LSTM-CNN-GBDT model with replacement recognition algorithm involved in φ-OTDR open source data of literature [10] are added for comparison, and the comparison of classification accuracy of seven algorithms is shown in Fig 7. Compared with GBDT, CatBoost can automatically process category features, reduce the cost of manual parameter adjustment, and usually has higher classification accuracy under the same data through gradient deviation correction and leaf node orderly splitting. The recognition accuracy of LSTM-CNN-CatBoost-GSSSA model reaches 97.6%. Compared with SVM, CNN, LSTM-CatBoost, CNN-CatBoost, LSTM-CNN-GBDT, and LSTM-CNN-CatBoost, the recognition accuracy is improved by 15%, 3.6%, 14.79%, 1.2%, 1.4%, 0.49%, respectively. It is proved that the LSTM-CNN-CatBoost-GSSSA model has a good effect on the recognition of the external broken source vibration signal of optical cable.

## 4. Conclusion

This study proposes an LSTM-CNN-CatBoost-GSSSA recognition method that integrates deep learning and ensemble learning to enhance the identification of external damage sources in underground ADSS optical cables within urban environments. By combining LSTM and CNN branches, the model extracts and fuses temporal, frequency, and spatial features. The layer dynamically adjusts the contribution of features from different domains through an adaptive weighting mechanism. The weight of spatial-frequency domain features is increased for external damage events (such as digging and knocking), while interference events (such as walking) rely more heavily on temporal sequence patterns. This approach effectively achieves information complementarity and redundancy suppression across multi-domain heterogeneous features. Finally, an improved GSSSA optimization algorithm is introduced. On one hand, feature weights are globally optimized using the enhanced golden sine operator to strengthen key discriminative features. On the other hand, the sparrow search algorithm’s swarm intelligence is employed to optimize the core hyperparameters of CatBoost in parallel. Its dynamic exploration and exploitation mechanism effectively prevents the traditional method’s tendency to become trapped in local optima, enabling the model to achieve a better decision boundary. The model reached 97.6% accuracy across six vibration events, exceeding benchmarks and confirming the advantage of integrating temporal features with GSSSA optimization. For practical operation and maintenance, this method enhances damage diagnosis efficiency and power communication network stability. A current limitation is the use of lab-simulated data, which may not represent extreme field scenarios, potentially affecting real-world accuracy. Future work will focus on scene expansion by gathering field vibration data to build a more realistic dataset and improve model adaptability.

## Supporting information

S1 File Code sharing declaration. (DOCX)
