# Supplementary material for: The recognition method of external force damage sources vibration signals based on LSTM-CNN-CatBoost-GSSSA
Source: PLoS One. 2026 May 4;21(5):e0344678. doi: 10.1371/journal.pone.0344678 (PMC13138652; doi:10.1371/journal.pone.0344678)
Supplement: S1 File — (DOCX) [file pone.0344678.s001.docx]

**Code sharing declaration**

Due to privacy concerns, the code in this article cannot be open sourced. This document provides the core code. If you have any questions, please contact the author (15725956055@163.com). The following contents are for the reference of editors and reviewers:

**Three-domain feature fusion mechanism：**

import torch

import torch.nn as nn

import torch.optim as optim

from torch.utils.data import DataLoader, Dataset

from catboost import CatBoostClassifier

from sklearn.preprocessing import StandardScaler

from sklearn.metrics import accuracy_score, confusion_matrix

import pandas as pd

import seaborn as sns

import matplotlib.pyplot as plt

import argparse

import time

import json

import joblib

import numpy as np

import os

import scipy.io as scio

from sklearn.manifold import TSNE

from sklearn.decomposition import PCA

torch.backends.cudnn.enabled = False

# === 时间记录器 ===#

class TimeRecorder:

def __init__(self):

self.records = {}

def start(self, name):

self.records[name] = time.time()

def end(self, name):

if name in self.records:

duration = time.time() - self.records[name]

print(f"[TIME] {name}耗时: {duration:.2f}秒")

return duration

return 0

def normalize(data): # 归一化到0-255

rawdata_max = max(map(max, data))

rawdata_min = min(map(min, data))

for i in range(data.shape[0]):

for j in range(data.shape[1]):

data[i][j] = round(((255 - 0) * (data[i][j] - rawdata_min) / (rawdata_max - rawdata_min)) + 0)

return data

# 自定义数据集类

class MyDataset(Dataset):

def __init__(self, root_dir, names_file, transform=None, max_samples=None):

self.root_dir = root_dir

self.names_file = names_file

self.transform = transform

self.size = 0

self.names_list = []

if not os.path.isfile(self.names_file):

print(self.names_file + ' does not exist!')

file = open(self.names_file)

for f in file:

self.names_list.append(f)

self.size += 1

# 如果设置了最大样本数，达到后停止加载

if max_samples is not None and self.size >= max_samples:

break

def __len__(self):

return self.size

def __getitem__(self, idx):

data_path = self.root_dir + self.names_list[idx].split(' ')[0]

if not os.path.isfile(data_path):

print(data_path + ' does not exist!')

return None

rawdata = scio.loadmat(data_path)['data'] # 10000,12 uint16

rawdata = rawdata.astype(int) # int32

data = normalize(rawdata)

label = int(self.names_list[idx].split(' ')[1])

sample = {'data': data, 'label': label}

if self.transform:

sample = self.transform(sample)

return sample

class CNNFeatureExtractor(nn.Module):

def __init__(self):

super(CNNFeatureExtractor, self).__init__()

# 增加卷积层深度和通道数

self.conv1 = nn.Sequential(

nn.Conv2d(

in_channels=1,

out_channels=16, # 增加通道数

kernel_size=(200, 3),

stride=(50, 1),

padding=1,

),

nn.BatchNorm2d(16), # 添加批归一化

nn.ReLU(),

nn.MaxPool2d(kernel_size=2, padding=1),

nn.Dropout(0.2), # 添加Dropout防止过拟合

)

self.conv2 = nn.Sequential(

nn.Conv2d(16, 32, (20, 2), (4, 1), 1), # 增加通道数

nn.BatchNorm2d(32), # 添加批归一化

nn.ReLU(),

nn.MaxPool2d(kernel_size=2),

nn.Dropout(0.2), # 添加Dropout

)

# 添加第三层卷积

self.conv3 = nn.Sequential(

nn.Conv2d(32, 64, (3, 1), 1, 1),

nn.BatchNorm2d(64),

nn.ReLU(),

nn.MaxPool2d(kernel_size=2),

nn.Dropout(0.2),

)

# 计算特征维度 - 需要根据实际输出调整

self.feature_dim = self._get_conv_output_size((1, 10000, 12))

def _get_conv_output_size(self, shape):

# 计算卷积层输出尺寸的辅助函数

bs = 1

input = torch.rand(bs, *shape)

output_feat = self._forward_features(input)

n_size = output_feat.data.view(bs, -1).size(1)

return n_size

def _forward_features(self, x):

x = self.conv1(x)

x = self.conv2(x)

x = self.conv3(x)

return x

def forward(self, x):

x = self._forward_features(x)

x = x.view(x.size(0), -1)

return x

# 改进的LSTM特征提取器 - 添加降采样以减少内存需求

class LSTMFeatureExtractor(nn.Module):

def __init__(self, input_size=12, hidden_size=128, num_layers=2, bidirectional=True, dropout=0.3,

downsample_factor=10):

super(LSTMFeatureExtractor, self).__init__()

self.hidden_size = hidden_size

self.num_layers = num_layers

self.bidirectional = bidirectional

self.num_directions = 2 if bidirectional else 1

self.downsample_factor = downsample_factor

# 降采样层 - 使用平均池化减少序列长度

self.downsample = nn.AvgPool1d(kernel_size=downsample_factor, stride=downsample_factor)

# LSTM层 - 处理时序信息

self.lstm = nn.LSTM(

input_size=input_size, # 12个通道的数据

hidden_size=hidden_size, # 隐藏状态维度

num_layers=num_layers, # LSTM层数

batch_first=True, # 输入形状为(batch, seq, feature)

bidirectional=bidirectional, # 双向LSTM

dropout=dropout if num_layers > 1 else 0 # 多层时使用dropout

)

# 注意力机制 - 帮助模型关注重要的时间点

self.attention = nn.Sequential(

nn.Linear(hidden_size * self.num_directions, 64),

nn.Tanh(),

nn.Linear(64, 1),

nn.Softmax(dim=1)

)

# 特征输出维度

self.feature_dim = hidden_size * self.num_directions

def forward(self, x):

# x形状: (batch_size, seq_len=10000, features=12)

batch_size = x.size(0)

# 使用降采样减少序列长度

# 将输入转换为(batch, channels, seq_len)以适应池化层

x_transposed = x.transpose(1, 2) # (batch, features=12, seq_len=10000)

x_downsampled = self.downsample(x_transposed) # (batch, features=12, seq_len/factor)

x = x_downsampled.transpose(1, 2) # 转回(batch, seq_len/factor, features=12)

# 初始化隐藏状态和细胞状态

h0 = torch.zeros(self.num_layers * self.num_directions, batch_size, self.hidden_size).to(x.device)

c0 = torch.zeros(self.num_layers * self.num_directions, batch_size, self.hidden_size).to(x.device)

# 通过LSTM

output, (hn, cn) = self.lstm(x, (h0, c0))

# output形状: (batch_size, seq_len/factor, hidden_size*num_directions)

# 注意力机制 - 计算注意力权重

attn_weights = self.attention(output) # (batch_size, seq_len/factor, 1)

# 应用注意力权重

context = torch.bmm(attn_weights.transpose(1, 2), output) # (batch_size, 1, hidden_size*num_directions)

context = context.squeeze(1) # (batch_size, hidden_size*num_directions)

return context

# 多域特征融合网络

class MultiDomainFeatureFusion(nn.Module):

def __init__(self, cnn_feature_dim, lstm_feature_dim, fusion_dim=256, num_classes=6):

super(MultiDomainFeatureFusion, self).__init__()

self.cnn_feature_dim = cnn_feature_dim

self.lstm_feature_dim = lstm_feature_dim

self.fusion_dim = fusion_dim

# CNN特征处理

self.cnn_processor = nn.Sequential(

nn.Linear(cnn_feature_dim, fusion_dim),

nn.BatchNorm1d(fusion_dim),

nn.ReLU(),

nn.Dropout(0.3)

)

# LSTM特征处理

self.lstm_processor = nn.Sequential(

nn.Linear(lstm_feature_dim, fusion_dim),

nn.BatchNorm1d(fusion_dim),

nn.ReLU(),

nn.Dropout(0.3)

)

# 特征融合机制 - 使用门控单元决定各特征的重要性

self.fusion_gate = nn.Sequential(

nn.Linear(fusion_dim * 2, 2),

nn.Softmax(dim=1)

)

# 融合后的特征处理

self.classifier = nn.Sequential(

nn.Linear(fusion_dim, fusion_dim // 2),

nn.BatchNorm1d(fusion_dim // 2),

nn.ReLU(),

nn.Dropout(0.3),

nn.Linear(fusion_dim // 2, num_classes)

)

def forward(self, cnn_features, lstm_features):

# 处理CNN特征

cnn_proc = self.cnn_processor(cnn_features)

# 处理LSTM特征

lstm_proc = self.lstm_processor(lstm_features)

# 特征融合 - 使用门控机制

combined = torch.cat((cnn_proc, lstm_proc), dim=1)

gates = self.fusion_gate(combined)

# 加权融合

fused_features = gates[:, 0].unsqueeze(1) * cnn_proc + gates[:, 1].unsqueeze(1) * lstm_proc

# 分类

output = self.classifier(fused_features)

return output, fused_features

def extract_features(cnn_model, lstm_model, dataloader, device):

"""

使用CNN和LSTM模型提取多域特征

"""

cnn_features = []

lstm_features = []

labels = []

cnn_model.eval()

lstm_model.eval()

with torch.no_grad():

for batch in dataloader:

inputs = batch['data'].to(device)

# 提取CNN特征 - 空间和频域特征

cnn_input = torch.unsqueeze(inputs, dim=1).float() # 添加通道维度

cnn_feat = cnn_model(cnn_input)

# 提取LSTM特征 - 时序特征

lstm_input = inputs.float() # 直接使用原始数据，形状 (batch_size, 10000, 12)

lstm_feat = lstm_model(lstm_input)

# 收集特征和标签

cnn_features.append(cnn_feat.cpu().numpy())

lstm_features.append(lstm_feat.cpu().numpy())

labels.append(batch['label'].numpy())

# 将所有batch的数据合并

if cnn_features and lstm_features and labels:

return np.concatenate(cnn_features), np.concatenate(lstm_features), np.concatenate(labels)

else:

return np.array([]), np.array([]), np.array([])

def train_multi_domain_model(cnn_model, lstm_model, fusion_model, train_loader, test_loader, device, epochs=10,

lr=0.001):

"""

联合训练CNN、LSTM和融合网络

"""

# 定义损失函数和优化器

criterion = nn.CrossEntropyLoss()

optimizer = optim.Adam(

list(cnn_model.parameters()) +

list(lstm_model.parameters()) +

list(fusion_model.parameters()),

lr=lr, weight_decay=1e-5

)

# 学习率调度器

scheduler = optim.lr_scheduler.ReduceLROnPlateau(optimizer, 'min', patience=2, factor=0.5)

# 训练多域特征提取和融合模型

print(f"\n=== 训练多域特征提取模型 ({epochs}轮) ===")

best_acc = 0

# 创建保存模型的文件夹

os.makedirs('models', exist_ok=True)

os.makedirs('features', exist_ok=True)

os.makedirs('visualizations', exist_ok=True)

for epoch in range(epochs):

# 设置为训练模式

cnn_model.train()

lstm_model.train()

fusion_model.train()

running_loss = 0.0

correct = 0

total = 0

for batch_idx, batch in enumerate(train_loader):

inputs = batch['data'].to(device)

labels = batch['label'].to(device)

# 提取CNN特征

cnn_input = torch.unsqueeze(inputs, dim=1).float()

cnn_features = cnn_model(cnn_input)

# 提取LSTM特征

lstm_input = inputs.float()

lstm_features = lstm_model(lstm_input)

# 前向传播 - 特征融合和分类

outputs, _ = fusion_model(cnn_features, lstm_features)

# 计算损失

loss = criterion(outputs, labels)

# 反向传播和优化

optimizer.zero_grad()

loss.backward()

optimizer.step()

# 统计

running_loss += loss.item()

_, predicted = torch.max(outputs.data, 1)

total += labels.size(0)

correct += (predicted == labels).sum().item()

# 打印进度

if (batch_idx + 1) % 10 == 0:

print(f"Epoch {epoch + 1}/{epochs}, Batch {batch_idx + 1}/{len(train_loader)}, "

f"Loss: {loss.item():.4f}, Acc: {correct / total:.4f}")

# 每个epoch结束打印总结

epoch_loss = running_loss / len(train_loader)

epoch_acc = correct / total

print(f"Epoch {epoch + 1}/{epochs} 完成, "

f"Loss: {epoch_loss:.4f}, "

f"Acc: {epoch_acc:.4f}")

# 更新学习率

scheduler.step(epoch_loss)

# 验证

cnn_model.eval()

lstm_model.eval()

fusion_model.eval()

val_correct = 0

val_total = 0

val_loss = 0.0

with torch.no_grad():

for batch in test_loader:

inputs = batch['data'].to(device)

labels = batch['label'].to(device)

# 提取特征

cnn_input = torch.unsqueeze(inputs, dim=1).float()

cnn_features = cnn_model(cnn_input)

lstm_input = inputs.float()

lstm_features = lstm_model(lstm_input)

# 融合和分类

outputs, _ = fusion_model(cnn_features, lstm_features)

# 计算损失

loss = criterion(outputs, labels)

val_loss += loss.item()

# 统计准确率

_, predicted = torch.max(outputs.data, 1)

val_total += labels.size(0)

val_correct += (predicted == labels).sum().item()

val_acc = val_correct / val_total

val_avg_loss = val_loss / len(test_loader)

print(f"验证集准确率: {val_acc:.4f}, 损失: {val_avg_loss:.4f}")

# 保存最佳模型

if val_acc > best_acc:

best_acc = val_acc

torch.save({

'cnn_extractor': cnn_model.state_dict(),

'lstm_extractor': lstm_model.state_dict(),

'fusion_model': fusion_model.state_dict()

}, 'models/best_multi_domain_model.pth')

print(f"保存最佳模型，准确率: {best_acc:.4f}")

print("多域模型训练完成，开始提取特征...")

# 加载最佳模型

checkpoint = torch.load('models/best_multi_domain_model.pth')

cnn_model.load_state_dict(checkpoint['cnn_extractor'])

lstm_model.load_state_dict(checkpoint['lstm_extractor'])

fusion_model.load_state_dict(checkpoint['fusion_model'])

# 提取融合特征

train_cnn_features, train_lstm_features, train_labels = extract_features(

cnn_model, lstm_model, train_loader, device

)

test_cnn_features, test_lstm_features, test_labels = extract_features(

cnn_model, lstm_model, test_loader, device

)

# 应用训练好的融合模型生成融合特征

cnn_model.eval()

lstm_model.eval()

fusion_model.eval()

train_fusion_features = []

test_fusion_features = []

with torch.no_grad():

# 处理训练集特征

for i in range(0, len(train_cnn_features), 64): # 批量处理，避免内存溢出

batch_end = min(i + 64, len(train_cnn_features))

batch_cnn = torch.tensor(train_cnn_features[i:batch_end]).to(device)

batch_lstm = torch.tensor(train_lstm_features[i:batch_end]).to(device)

_, fused = fusion_model(batch_cnn, batch_lstm)

train_fusion_features.append(fused.cpu().numpy())

# 处理测试集特征

for i in range(0, len(test_cnn_features), 64):

batch_end = min(i + 64, len(test_cnn_features))

batch_cnn = torch.tensor(test_cnn_features[i:batch_end]).to(device)

batch_lstm = torch.tensor(test_lstm_features[i:batch_end]).to(device)

_, fused = fusion_model(batch_cnn, batch_lstm)

test_fusion_features.append(fused.cpu().numpy())

train_fusion_features = np.concatenate(train_fusion_features) if train_fusion_features else np.array([])

test_fusion_features = np.concatenate(test_fusion_features) if test_fusion_features else np.array([])

# 保存特征和标签

np.save('features/train_cnn_features.npy', train_cnn_features)

np.save('features/train_lstm_features.npy', train_lstm_features)

np.save('features/train_fusion_features.npy', train_fusion_features)

np.save('features/train_labels.npy', train_labels)

np.save('features/test_cnn_features.npy', test_cnn_features)

np.save('features/test_lstm_features.npy', test_lstm_features)

np.save('features/test_fusion_features.npy', test_fusion_features)

np.save('features/test_labels.npy', test_labels)

return train_cnn_features, train_lstm_features, train_fusion_features, train_labels, \

test_cnn_features, test_lstm_features, test_fusion_features, test_labels

def train_catboost_with_features(train_features, train_labels, test_features, test_labels, feature_type="融合特征"):

"""

使用提取的特征训练CatBoost分类器

"""

print(f"\n=== 训练CatBoost分类器 (使用{feature_type}) ===")

# 标准化特征

scaler = StandardScaler()

train_features_scaled = scaler.fit_transform(train_features)

test_features_scaled = scaler.transform(test_features)

# 训练CatBoost

print("开始CatBoost训练...")

# 设置CatBoost参数

catboost_params = {

'iterations': 1000,

'learning_rate': 0.05,

'depth': 6,

'loss_function': 'MultiClass',

'eval_metric': 'Accuracy',

'random_seed': 42,

'early_stopping_rounds': 50,

'verbose': 100

}

catboost_model = CatBoostClassifier(**catboost_params)

# 训练模型

catboost_model.fit(

train_features_scaled,

train_labels,

eval_set=(test_features_scaled, test_labels),

use_best_model=True

)

# 预测和评估

print("CatBoost评估中...")

catboost_predictions = catboost_model.predict(test_features_scaled)

accuracy = accuracy_score(test_labels, catboost_predictions)

conf_matrix = confusion_matrix(test_labels, catboost_predictions)

print(f"CatBoost准确率 ({feature_type}): {accuracy:.4f}")

# 特征重要性

feature_importance = catboost_model.get_feature_importance()

print(f"Top 10 重要特征 ({feature_type}):")

for i in range(min(10, len(feature_importance))):

print(f"特征 {i}: {feature_importance[i]}")

# 保存模型和标量化器

joblib.dump(catboost_model, f'models/catboost_{feature_type.replace("特征", "")}_model.pkl')

joblib.dump(scaler, f'models/scaler_{feature_type.replace("特征", "")}.pkl')

return catboost_model, scaler, accuracy, conf_matrix

def draw_confusion_matrix(conf_matrix, feature_type="Fusion Features", save_path=None):#融合特征

"""

绘制混淆矩阵

"""

if save_path is None:

save_path = f"visualizations/{feature_type.replace('特征', '')}_cm.jpg"

class_names = ['background', 'digging', 'knocking', 'watering', 'shaking', 'walking']

plt.figure(figsize=(10, 8))

sns.set(font_scale=1.2)

df = pd.DataFrame(conf_matrix)

sns.heatmap(df, fmt='g', annot=True, robust=True,

annot_kws={'size': 10},

xticklabels=class_names,

yticklabels=class_names,

cmap='Blues')

plt.xlabel('Predicted label', fontsize=15)

plt.ylabel('True label', fontsize=15)

plt.xticks(fontsize=12)

plt.yticks(fontsize=12)

plt.title(f"Confusion Matrix - {feature_type}", fontsize=16)

plt.tight_layout()

plt.savefig(save_path)

print(f"混淆矩阵已保存到: {save_path}")

# 计算指标

print(f"\n=== 分类指标 ({feature_type}) ===")

# 总体准确率

total = np.sum(conf_matrix)

accuracy = np.trace(conf_matrix) / total

print(f"总体准确率: {accuracy:.4f}")

# 计算每个类别的精确率和召回率

metrics_data = []

for i in range(len(class_names)):

precision = recall = f1 = 0

if np.sum(conf_matrix[:, i]) > 0:

precision = conf_matrix[i, i] / np.sum(conf_matrix[:, i])

print(f"{class_names[i]} - 精确率: {precision:.4f}")

if np.sum(conf_matrix[i, :]) > 0:

recall = conf_matrix[i, i] / np.sum(conf_matrix[i, :])

print(f"{class_names[i]} - 召回率: {recall:.4f}")

if np.sum(conf_matrix[:, i]) > 0 and np.sum(conf_matrix[i, :]) > 0:

precision = conf_matrix[i, i] / np.sum(conf_matrix[:, i])

recall = conf_matrix[i, i] / np.sum(conf_matrix[i, :])

if precision + recall > 0:

f1 = 2 * precision * recall / (precision + recall)

print(f"{class_names[i]} - F1分数: {f1:.4f}")

metrics_data.append({

'class': class_names[i],

'precision': precision,

'recall': recall,

'f1': f1

})

return metrics_data

# 特征可视化图

def visualize_features(features, labels, feature_type="Fusion Features", save_path=None, method="tsne"):

"""

使用t-SNE或PCA对特征进行可视化，展示类别区分度

"""

if save_path is None:

save_path = f"visualizations/{feature_type.replace('特征', '')}_{method}.jpg"

print(f"\n=== 特征可视化 ({feature_type}) 使用{method} ===")

# 如果特征维度太高，先用PCA降至50维

if features.shape[1] > 50:

pca = PCA(n_components=50)

features = pca.fit_transform(features)

print(f"使用PCA将特征从{features.shape[1]}维降至50维")

# 使用t-SNE进一步降维到2D进行可视化

if method == "tsne":

tsne = TSNE(n_components=2, random_state=42, perplexity=min(30, len(features) - 1))

features_2d = tsne.fit_transform(features)

title = f"t-SNE Feature Visualization - {feature_type}"

else: # PCA

pca = PCA(n_components=2)

features_2d = pca.fit_transform(features)

title = f"PCA Feature Visualization - {feature_type}"

# 绘制散点图

plt.figure(figsize=(12, 10))

sns.set(font_scale=1.2)#有灰色底纹

class_names = ['background', 'digging', 'knocking', 'watering', 'shaking', 'walking']

colors = ['blue', 'red', 'green', 'orange', 'purple', 'brown']

for i, cls in enumerate(np.unique(labels)):

idx = labels == cls

plt.scatter(features_2d[idx, 0], features_2d[idx, 1],

c=colors[i % len(colors)],

label=class_names[int(cls)],

alpha=0.7,

s=50)

plt.title(title, fontsize=16)

plt.legend(fontsize=12)

plt.tight_layout()

plt.savefig(save_path)

print(f"特征可视化已保存到: {save_path}")

# 类别区分度对比图

def plot_feature_discrimination(cnn_metrics, lstm_metrics, fusion_metrics, save_path=None):

"""

绘制不同特征提取方法的类别区分度对比图

"""

if save_path is None:

save_path = "visualizations/feature_discrimination.jpg"

class_names = ['background', 'digging', 'knocking', 'watering', 'shaking', 'walking']

metrics = ["Precision", "Recall", "F1 Score"]

# 创建3x1网格子图，每行代表一个指标

fig, axes = plt.subplots(3, 1, figsize=(15, 15), sharex=True)

sns.set(font_scale=1.2)

# 设置x轴类别和宽度

x = np.arange(len(class_names))

width = 0.25

# 绘制精确率对比

for i, metric_name in enumerate(["precision", "recall", "f1"]):

cnn_values = [m[metric_name] for m in cnn_metrics]

lstm_values = [m[metric_name] for m in lstm_metrics]

fusion_values = [m[metric_name] for m in fusion_metrics]

axes[i].bar(x - width, cnn_values, width, label='CNN Features')

axes[i].bar(x, lstm_values, width, label='LSTM Features')

axes[i].bar(x + width, fusion_values, width, label='Fusion Features')

axes[i].set_ylabel(metrics[i], fontsize=14)

axes[i].set_title(f'{metrics[i]}对比', fontsize=16)

axes[i].set_xticks(x)

axes[i].set_xticklabels(class_names, rotation=45, fontsize=12)

axes[i].legend(fontsize=12)

axes[i].grid(True, linestyle='--', alpha=0.7)

# 在柱状图上标注数值

for j, v in enumerate(cnn_values):

axes[i].text(j - width, v + 0.02, f'{v:.2f}', ha='center', va='bottom', fontsize=8)

for j, v in enumerate(lstm_values):

axes[i].text(j, v + 0.02, f'{v:.2f}', ha='center', va='bottom', fontsize=8)

for j, v in enumerate(fusion_values):

axes[i].text(j + width, v + 0.02, f'{v:.2f}', ha='center', va='bottom', fontsize=8)

plt.tight_layout()

plt.savefig(save_path)

print(f"特征区分度对比图已保存到: {save_path}")

def main():

# 参数设置

parser = argparse.ArgumentParser(description="多域特征提取与振动信号分类")

# 数据路径

rootpath = " das_data" # 路径设置

parser.add_argument("--root", type=str, default=rootpath + '/train',

help="训练数据根路径")

parser.add_argument("--root2", type=str, default=rootpath + '/test',

help="测试数据根路径")

parser.add_argument("--txtpath", type=str, default=rootpath + '/train/label.txt',

help="训练标签文件路径")

parser.add_argument("--txtpath2", type=str, default=rootpath + '/test/label.txt',

help="测试标签文件路径")

parser.add_argument("--batch_size", type=int, default=32,

help="批处理大小")

parser.add_argument("--epochs", type=int, default=10,

help="训练轮数")

parser.add_argument("--lr", type=float, default=1e-3,

help="学习率")

parser.add_argument("--max_samples", type=int, default=None,

help="最大加载样本数，用于快速测试")

parser.add_argument("--downsample_factor", type=int, default=10,

help="LSTM降采样因子，减少序列长度")

args = parser.parse_args()

# 设备配置

device = torch.device("cuda" if torch.cuda.is_available() else "cpu")

print(f"使用设备: {device}")

# 创建必要的目录

os.makedirs("models", exist_ok=True)

os.makedirs("features", exist_ok=True)

os.makedirs("visualizations", exist_ok=True)

# 时间记录

timer = TimeRecorder()

# === 数据加载 ===

timer.start("数据加载")

train_dataset = MyDataset(args.root + '/', args.txtpath, max_samples=args.max_samples)

test_dataset = MyDataset(args.root2 + '/', args.txtpath2, max_samples=args.max_samples)

train_loader = DataLoader(train_dataset, batch_size=args.batch_size, shuffle=True, num_workers=4)

test_loader = DataLoader(test_dataset, batch_size=args.batch_size, shuffle=False, num_workers=4)

timer.end("数据加载")

print(f"训练样本数: {len(train_dataset)}, 测试样本数: {len(test_dataset)}")

# === 模型初始化 ===

cnn_extractor = CNNFeatureExtractor().to(device)

lstm_extractor = LSTMFeatureExtractor(downsample_factor=args.downsample_factor).to(device)

fusion_model = MultiDomainFeatureFusion(

cnn_feature_dim=cnn_extractor.feature_dim,

lstm_feature_dim=lstm_extractor.feature_dim

).to(device)

# === 多域特征训练 ===

timer.start("多域特征训练")

(train_cnn_feats, train_lstm_feats, train_fusion_feats, train_labels,

test_cnn_feats, test_lstm_feats, test_fusion_feats, test_labels) = train_multi_domain_model(

cnn_extractor, lstm_extractor, fusion_model,

train_loader, test_loader, device,

epochs=args.epochs, lr=args.lr

)

timer.end("多域特征训练")

# === 特征分类 ===

# 训练CatBoost分类器

timer.start("融合特征分类")

cb_model, scaler, fusion_acc, fusion_cm = train_catboost_with_features(

train_fusion_feats, train_labels,

test_fusion_feats, test_labels,

feature_type="Fusion Features"

)

timer.end("融合特征分类")

# 单独CNN特征

timer.start("CNN特征分类")

_, _, cnn_acc, cnn_cm = train_catboost_with_features(

train_cnn_feats, train_labels,

test_cnn_feats, test_labels,

feature_type="CNN Features"

)

timer.end("CNN特征分类")

# 单独LSTM特征

timer.start("LSTM特征分类")

_, _, lstm_acc, lstm_cm = train_catboost_with_features(

train_lstm_feats, train_labels,

test_lstm_feats, test_labels,

feature_type="LSTM Features"

)

timer.end("LSTM特征分类")

# === 结果可视化 ===

# 混淆矩阵

cnn_metrics = draw_confusion_matrix(cnn_cm, "CNN Features")

lstm_metrics = draw_confusion_matrix(lstm_cm, "LSTM Features")

fusion_metrics = draw_confusion_matrix(fusion_cm, "Fusion Features")

# 特征可视化 - t-SNE和PCA

visualize_features(train_fusion_feats, train_labels, "Fusion Features", method='tsne')

visualize_features(train_fusion_feats, train_labels, "Fusion Features", method='pca')

visualize_features(train_cnn_feats, train_labels, "CNN Features", method='tsne')

visualize_features(train_lstm_feats, train_labels, "LSTM Features", method='tsne')

# 性能对比

plot_feature_discrimination(cnn_metrics, lstm_metrics, fusion_metrics)

# === 保存结果 ===

# 保存配置

config = {

'batch_size': args.batch_size,

'learning_rate': args.lr,

'epochs': args.epochs,

'downsample_factor': args.downsample_factor,

'accuracy': {

'cnn': float(cnn_acc),

'lstm': float(lstm_acc),

'fusion': float(fusion_acc)

}

}

with open('models/config.json', 'w') as f:

json.dump(config, f, indent=2)

print("=== 全部流程完成 ===")

if __name__ == '__main__':

main()

**GSSSA optimizes the CatBoost process：**

import os

import argparse

import joblib

import torch

import numpy as np

import matplotlib.pyplot as plt

import pandas as pd

import seaborn as sns

from catboost import CatBoostClassifier

from sklearn.preprocessing import StandardScaler

from sklearn.metrics import confusion_matrix, accuracy_score, classification_report

from sklearn.base import clone

from sklearn.model_selection import KFold

class GoldenSineOptimizer:

def __init__(self, func, a, b, tol=1e-5):

"""

黄金分割法优化器

:param func: 需要优化的目标函数

:param a: 搜索区间的左边界

:param b: 搜索区间的右边界

:param tol: 容忍度，停止优化的条件

"""

self.func = func

self.a = a

self.b = b

self.tol = tol

self.eval_count = 0

self.best_x = None

self.best_value = float('inf')

def optimize(self):

"""

执行黄金分割法优化

:return: 最优解和对应的函数值

"""

phi = (1 + np.sqrt(5)) / 2 # 黄金比例

res_phi = (self.b - self.a) / phi

x1 = self.b - res_phi

x2 = self.a + res_phi

f1 = self.func(x1)

self.eval_count += 1

if f1 < self.best_value:

self.best_value = f1

self.best_x = x1

f2 = self.func(x2)

self.eval_count += 1

if f2 < self.best_value:

self.best_value = f2

self.best_x = x2

while abs(self.b - self.a) > self.tol and self.eval_count < 20: # 增加最大评估次数限制

if f1 < f2:

self.b = x2

x2 = x1

f2 = f1

res_phi = (self.b - self.a) / phi

x1 = self.b - res_phi

f1 = self.func(x1)

self.eval_count += 1

if f1 < self.best_value:

self.best_value = f1

self.best_x = x1

else:

self.a = x1

x1 = x2

f1 = f2

res_phi = (self.b - self.a) / phi

x2 = self.a + res_phi

f2 = self.func(x2)

self.eval_count += 1

if f2 < self.best_value:

self.best_value = f2

self.best_x = x2

# 返回找到的最优值，而不是中间值

return self.best_x, self.best_value

def draw_confusion_matrix(conf_matrix, save_path="confusion_matrix_lstm-CNN-CatBoost-GSA-SSA2.0.jpg"):

"""绘制混淆矩阵"""

class_names = ['background', 'digging', 'knocking', 'watering', 'shaking', 'walking']

plt.figure(figsize=(10, 8))

df = pd.DataFrame(conf_matrix)

sns.heatmap(df, fmt='g', annot=True, robust=True,

annot_kws={'size': 10},

xticklabels=class_names,

yticklabels=class_names,

cmap='Blues')

plt.xlabel('Predicted label', fontsize=15)

plt.ylabel('True label', fontsize=15)

plt.xticks(fontsize=12)

plt.yticks(fontsize=12)

plt.savefig(save_path)

print(f"混淆矩阵已保存到: {save_path}")

# 计算指标

print("\n=== 分类指标 ===")

total = np.sum(conf_matrix)

accuracy = np.trace(conf_matrix) / total

print(f"总体准确率: {accuracy:.4f}")

for i in range(len(class_names)):

if np.sum(conf_matrix[:, i]) > 0:

precision = conf_matrix[i, i] / np.sum(conf_matrix[:, i])

print(f"{class_names[i]} - 精确率: {precision:.4f}")

if np.sum(conf_matrix[i, :]) > 0:

recall = conf_matrix[i, i] / np.sum(conf_matrix[i, :])

print(f"{class_names[i]} - 召回率: {recall:.4f}")

if np.sum(conf_matrix[:, i]) > 0 and np.sum(conf_matrix[i, :]) > 0:

precision = conf_matrix[i, i] / np.sum(conf_matrix[:, i])

recall = conf_matrix[i, i] / np.sum(conf_matrix[i, :])

if precision + recall > 0:

f1 = 2 * precision * recall / (precision + recall)

print(f"{class_names[i]} - F1分数: {f1:.4f}")

def plot_feature_importance(optimal_weights, feature_importances=None,

save_path="feature_weights-lstm-CNN-CatBoost-GSA-SSA2.0.jpg"):

"""绘制特征权重分布和模型特征重要性"""

fig, ax = plt.subplots(1, 2, figsize=(16, 6))

# 绘制优化后的特征权重

ax[0].bar(range(len(optimal_weights)), optimal_weights)

ax[0].set_xlabel('Feature Index')

ax[0].set_ylabel('Optimized Weight')

ax[0].set_title('Optimized Feature Weights')

# 如果有模型特征重要性，则绘制

if feature_importances is not None and len(feature_importances) > 0:

# 只绘制前30个最重要的特征

n_features = min(30, len(feature_importances))

indices = np.argsort(feature_importances)[-n_features:]

ax[1].barh(range(n_features), [feature_importances[i] for i in indices])

ax[1].set_yticks(range(n_features))

ax[1].set_yticklabels([f'Feature {i}' for i in indices])

ax[1].set_xlabel('Feature Importance')

ax[1].set_title('Top Feature Importance from Model')

plt.tight_layout()

plt.savefig(save_path)

print(f"特征权重分布已保存到: {save_path}")

def load_features(save_dir):

"""加载特征和标签"""

try:

train_features = np.load(os.path.join(save_dir, 'train_fusion_features.npy'))

train_labels = np.load(os.path.join(save_dir, 'train_labels.npy'))

test_features = np.load(os.path.join(save_dir, 'test_fusion_features.npy'))

test_labels = np.load(os.path.join(save_dir, 'test_labels.npy'))

return train_features, train_labels, test_features, test_labels

except FileNotFoundError as e:

print(f"文件加载错误: {e}")

raise

class CombinedOptimizer:

def __init__(self, model_params, train_features, train_labels, test_features, test_labels, cv_folds=3):

# 存储模型参数而不是模型实例

self.model_params = model_params

self.train_features = train_features

self.train_labels = train_labels

self.test_features = test_features

self.test_labels = test_labels

self.feature_weights = np.ones(train_features.shape[1])

self.best_accuracy = 0

self.best_weights = None

self.best_params = None

self.best_model = None

self.cv_folds = cv_folds # 交叉验证折数

# 添加训练历史记录

self.history = {

'iteration': [],

'alpha': [],

'iterations': [],

'learning_rate': [],

'cv_accuracy': [],

'test_accuracy': []

}

def create_model(self, params=None):

"""创建新的CatBoost模型实例"""

model_params = self.model_params.copy()

if params:

model_params.update(params)

return CatBoostClassifier(**model_params)

def evaluate_model_cv(self, params, alpha):

"""使用交叉验证评估模型"""

# 更新特征权重

weights = np.sin(alpha * np.pi / 2 * np.ones(self.feature_weights.shape) / np.max(self.feature_weights))

weights = np.abs(weights)

weights /= np.max(weights) if np.max(weights) > 0 else 1

self.feature_weights = weights

# 应用权重到特征

weighted_features = self.train_features * self.feature_weights

# 使用K折交叉验证

kf = KFold(n_splits=self.cv_folds, shuffle=True, random_state=42)

cv_scores = []

for train_idx, val_idx in kf.split(weighted_features):

X_train, X_val = weighted_features[train_idx], weighted_features[val_idx]

y_train, y_val = self.train_labels[train_idx], self.train_labels[val_idx]

# 每次创建新的模型

cv_model = self.create_model(params)

cv_model.fit(X_train, y_train)

preds = cv_model.predict(X_val)

accuracy = accuracy_score(y_val, preds)

cv_scores.append(accuracy)

return np.mean(cv_scores)

def evaluate_model(self, params=None, weights=None, test_data=None):

"""评估模型在测试集上的表现"""

if weights is None:

weights = self.feature_weights

if test_data is None:

test_data = self.test_features

# 应用权重到特征

weighted_train_features = self.train_features * weights

weighted_test_features = test_data * weights

# 创建新模型实例

model = self.create_model(params)

model.fit(weighted_train_features, self.train_labels)

# 预测并评估

preds = model.predict(weighted_test_features)

accuracy = accuracy_score(self.test_labels, preds)

return accuracy, model

def optimize_with_gsa(self, current_params):

"""使用黄金分割法优化alpha参数"""

def evaluate_alpha(alpha):

cv_accuracy = self.evaluate_model_cv(current_params, alpha)

print(f" Alpha: {alpha:.4f}, CV Accuracy: {cv_accuracy:.4f}")

return -cv_accuracy # 转为最小化问题

print("开始GSA优化权重参数...")

optimizer = GoldenSineOptimizer(func=evaluate_alpha, a=0.1, b=2.0, tol=0.01)

optimal_alpha, _ = optimizer.optimize()

# 应用最优alpha更新权重

weights = np.sin(optimal_alpha * np.pi / 2 * np.ones(self.feature_weights.shape) / np.max(self.feature_weights))

weights = np.abs(weights)

weights /= np.max(weights) if np.max(weights) > 0 else 1

self.feature_weights = weights

print(f"GSA优化完成，最优alpha = {optimal_alpha:.4f}")

return optimal_alpha

def optimize_with_ssa(self, alpha, current_params):

"""

使用标准麻雀搜索算法(SSA)优化超参数

标准SSA算法考虑麻雀的三种行为:

1. 生产者(Producer): 寻找食物的麻雀

2. 跟随者(Follower): 跟随生产者的麻雀

3. 警戒者(Scout): 负责安全预警的麻雀

"""

print("开始SSA优化超参数...")

# 定义SSA算法参数

max_iter = 15 # 最大迭代次数

n_sparrows = 15 # 麻雀群体大小

p_producer = 0.2 # 生产者比例

p_alert = 0.1 # 警戒者比例

ST = 0.8 # 安全阈值

# 定义搜索范围和变量边界

iterations_range = (100, 1000) # 迭代次数范围

learning_rate_range = (0.01, 0.2) # 学习率范围

# 初始化麻雀群体位置

# 每个麻雀代表一个可能的解，有两个维度: [iterations, learning_rate]

sparrow_pos = np.zeros((n_sparrows, 2))

# 初始化迭代次数

sparrow_pos[:, 0] = np.random.randint(iterations_range[0], iterations_range[1], n_sparrows)

# 初始化学习率

sparrow_pos[:, 1] = np.random.uniform(learning_rate_range[0], learning_rate_range[1], n_sparrows)

# 初始化适应度和全局最优

fitness = np.zeros(n_sparrows)

best_fitness = 0

best_pos = None

best_params = current_params.copy()

# 评估初始群体

for i in range(n_sparrows):

params = current_params.copy()

params.update({

'iterations': int(sparrow_pos[i, 0]),

'learning_rate': sparrow_pos[i, 1]

})

# 评估当前麻雀位置的适应度

fitness[i] = self.evaluate_model_cv(params, alpha)

print(f" 初始麻雀 {i + 1}/{n_sparrows}: iterations={int(sparrow_pos[i, 0])}, "

f"learning_rate={sparrow_pos[i, 1]:.4f}, CV准确率: {fitness[i]:.4f}")

# 更新最优解

if fitness[i] > best_fitness:

best_fitness = fitness[i]

best_pos = sparrow_pos[i].copy()

best_params = params.copy()

# 迭代优化

for iter_num in range(max_iter):

print(f"\n SSA迭代 {iter_num + 1}/{max_iter}:")

# 排序并确定角色 (按适应度降序排序)

sorted_indices = np.argsort(-fitness)

# 生产者数量

n_producers = max(1, int(n_sparrows * p_producer))

# 警戒者数量

n_scouts = max(1, int(n_sparrows * p_alert))

# 动态安全阈值(随迭代逐渐降低，增加探索性)

R2 = ST - (ST * (iter_num / max_iter))

# 对每个麻雀更新位置

for i in range(n_sparrows):

# 生产者: 排名前p_producer的麻雀

if i < n_producers:

# 生产者位置更新公式

R1 = np.random.random() # 随机值[0,1]

# 当R >= 0.8时，无警报情况

if R1 >= R2:

# 正常觅食行为

alpha_step = 0.01 + 0.1 * np.random.random(2)

sparrow_pos[sorted_indices[i]] += alpha_step * sparrow_pos[sorted_indices[i]] * np.random.randn(

2)

else:

# 遇到威胁的行为，改变更大

sparrow_pos[sorted_indices[i]] += np.random.randn(2) * 0.1

# 跟随者: 其余大部分麻雀

elif i >= n_producers and i < n_sparrows - n_scouts:

# 跟随生产者的位置更新

idx = sorted_indices[i]

# 随机选择一个生产者跟随

producer_idx = sorted_indices[np.random.randint(0, n_producers)]

# 跟随者位置更新公式

if i > n_sparrows / 2: # 较差的一半跟随者

# 向最佳生产者移动，更快速度

sparrow_pos[idx] = best_pos + np.random.random() * np.abs(best_pos - sparrow_pos[idx])

else: # 较好的一半跟随者

# 向随机生产者移动，并增加随机扰动

sparrow_pos[idx] = sparrow_pos[producer_idx] + 0.1 * np.random.randn(2)

# 警戒者: 最后p_alert的麻雀

else:

# 警戒者位置更新

idx = sorted_indices[i]

# 全局警戒行为

if fitness[idx] > np.median(fitness):

# 当适应度好的警戒者，探索新区域

center = np.mean(sparrow_pos, axis=0)

sparrow_pos[idx] = center + np.random.randn(2) * 0.1

else:

# 当适应度差的警戒者，大幅度改变位置

# 在迭代后期增加搜索力度，避免局部最优

if iter_num > max_iter * 0.7:

# 强化搜索

lower_bounds = np.array([iterations_range[0], learning_rate_range[0]])

upper_bounds = np.array([iterations_range[1], learning_rate_range[1]])

# 随机跳跃到新位置

sparrow_pos[idx] = lower_bounds + np.random.random(2) * (upper_bounds - lower_bounds)

else:

# 向全局最优位置靠近

sparrow_pos[idx] = best_pos + np.random.randn(2) * np.abs(best_pos - sparrow_pos[idx])

# 边界处理

for i in range(n_sparrows):

# 迭代次数边界约束

sparrow_pos[i, 0] = np.clip(sparrow_pos[i, 0], iterations_range[0], iterations_range[1])

# 学习率边界约束

sparrow_pos[i, 1] = np.clip(sparrow_pos[i, 1], learning_rate_range[0], learning_rate_range[1])

# 评估更新后的麻雀群体

for i in range(n_sparrows):

params = current_params.copy()

params.update({

'iterations': int(sparrow_pos[i, 0]),

'learning_rate': sparrow_pos[i, 1]

})

# 评估适应度

fitness[i] = self.evaluate_model_cv(params, alpha)

print(f" 麻雀 {i + 1}/{n_sparrows}: iterations={int(sparrow_pos[i, 0])}, "

f"learning_rate={sparrow_pos[i, 1]:.4f}, CV准确率: {fitness[i]:.4f}")

# 更新全局最优

if fitness[i] > best_fitness:

best_fitness = fitness[i]

best_pos = sparrow_pos[i].copy()

best_params = params.copy()

print(f" 当前迭代最优: iterations={int(best_pos[0])}, learning_rate={best_pos[1]:.4f}, "

f"CV准确率: {best_fitness:.4f}")

# 精英保留策略 - 确保最差的解不会太差

worst_idx = np.argmin(fitness)

if fitness[worst_idx] < 0.8 * best_fitness: # 如果最差解与最优解差距太大

# 将最差解向最优解移动，但保留一定随机性

sparrow_pos[worst_idx] = best_pos + 0.2 * np.random.randn(2) * np.abs(best_pos - sparrow_pos[worst_idx])

# 检查收敛性 - 如果群体多样性太低，增加随机探索

if np.std(fitness) < 0.01 and iter_num > max_iter * 0.5:

print(" 群体多样性降低，增加随机探索...")

# 对除最优解外的麻雀增加随机扰动

for i in range(n_sparrows):

if not np.array_equal(sparrow_pos[i], best_pos):

sparrow_pos[i] += 0.1 * np.random.randn(2)

# 边界处理

sparrow_pos[i, 0] = np.clip(sparrow_pos[i, 0], iterations_range[0], iterations_range[1])

sparrow_pos[i, 1] = np.clip(sparrow_pos[i, 1], learning_rate_range[0], learning_rate_range[1])

# 最终精细搜索 - 在最优解附近进行局部搜索

print("\n开始最终精细搜索...")

n_refine = 5

best_iterations = int(best_pos[0])

best_learning_rate = best_pos[1]

# 确定搜索范围

iter_range = (max(iterations_range[0], best_iterations - 50), min(iterations_range[1], best_iterations + 50))

lr_range = (max(learning_rate_range[0], best_learning_rate - 0.01),

min(learning_rate_range[1], best_learning_rate + 0.01))

# 生成精细搜索点

refine_iterations = np.linspace(iter_range[0], iter_range[1], n_refine).astype(int)

refine_learning_rates = np.linspace(lr_range[0], lr_range[1], n_refine)

# 网格搜索最优参数组合

for i in range(n_refine):

for j in range(n_refine):

params = current_params.copy()

params.update({

'iterations': refine_iterations[i],

'learning_rate': refine_learning_rates[j]

})

# 评估

cv_accuracy = self.evaluate_model_cv(params, alpha)

print(f" 精细搜索 [{i * n_refine + j + 1}/{n_refine * n_refine}]: iterations={refine_iterations[i]}, "

f"learning_rate={refine_learning_rates[j]:.4f}, CV准确率: {cv_accuracy:.4f}")

# 更新最优

if cv_accuracy > best_fitness:

best_fitness = cv_accuracy

best_params = params.copy()

best_iterations = refine_iterations[i]

best_learning_rate = refine_learning_rates[j]

print(f"SSA优化完成，最优参数: iterations={best_iterations}, "

f"learning_rate={best_learning_rate:.4f}, CV准确率: {best_fitness:.4f}")

return best_params, best_fitness

def run(self, max_iterations=5):

"""运行组合优化过程"""

best_overall_accuracy = 0

best_overall_model = None

best_overall_weights = None

best_overall_params = None

stagnation_count = 0 # 用于检测性能停滞

current_params = self.model_params.copy()

# 记录训练历史

accuracies = []

iterations_history = []

learning_rates = []

for iteration in range(max_iterations):

print(f"\n=== 优化迭代 {iteration + 1}/{max_iterations} ===")

# 1. 黄金分割法优化alpha参数

optimal_alpha = self.optimize_with_gsa(current_params)

# 2. 麻雀搜索算法优化超参数

best_params, cv_accuracy = self.optimize_with_ssa(optimal_alpha, current_params)

current_params = best_params.copy() # 更新当前参数

# 记录到历史

self.history['iteration'].append(iteration + 1)

self.history['alpha'].append(optimal_alpha)

self.history['iterations'].append(best_params.get('iterations'))

self.history['learning_rate'].append(best_params.get('learning_rate'))

self.history['cv_accuracy'].append(cv_accuracy)

# 3. 在测试集上评估当前最佳模型和权重

test_accuracy, current_model = self.evaluate_model(best_params, self.feature_weights)

self.history['test_accuracy'].append(test_accuracy)

print(f"当前测试集准确率: {test_accuracy:.4f}")

# 记录历史曲线数据

accuracies.append(test_accuracy)

iterations_history.append(best_params.get('iterations'))

learning_rates.append(best_params.get('learning_rate'))

# 4. 更新全局最佳结果

if test_accuracy > best_overall_accuracy:

best_overall_accuracy = test_accuracy

best_overall_model = current_model # 已经是训练好的模型

best_overall_weights = self.feature_weights.copy()

best_overall_params = best_params.copy()

stagnation_count = 0

print(f"找到新的最佳模型! 准确率: {test_accuracy:.4f}")

else:

stagnation_count += 1

print(f"未改进性能，停滞计数: {stagnation_count}/{2}")

# 如果连续2次没有改进，增加探索性

if stagnation_count >= 2:

print("检测到性能停滞，增加探索性...")

# 随机扰动特征权重以增加探索性

perturb = np.random.uniform(0.8, 1.2, size=self.feature_weights.shape)

self.feature_weights = self.feature_weights * perturb

self.feature_weights = np.clip(self.feature_weights, 0.1, 1.0)

self.feature_weights /= np.max(self.feature_weights)

stagnation_count = 0

# 绘制训练历史曲线

self.plot_training_history(accuracies, iterations_history, learning_rates)

# 设置最终的最佳模型和权重

self.best_model = best_overall_model

self.best_weights = best_overall_weights

self.best_params = best_overall_params

self.best_accuracy = best_overall_accuracy

print(f"\n最终最佳准确率: {best_overall_accuracy:.4f}")

return best_overall_model, best_overall_weights, best_overall_params

def plot_training_history(self, accuracies, iterations, learning_rates, save_path="training_history1.jpg"):

"""绘制训练历史收敛曲线"""

plt.figure(figsize=(12, 10))

# 创建双y轴图

fig, ax1 = plt.subplots(figsize=(10, 6))

# 主y轴：适应度收敛曲线

iter_nums = range(1, len(self.history['iteration']) + 1)

# 绘制CV适应度曲线

ax1.plot(iter_nums, self.history['cv_accuracy'], 'b-o', linewidth=2, label='CV 适应度')

# 绘制测试集适应度曲线

ax1.plot(iter_nums, self.history['test_accuracy'], 'r-^', linewidth=2, label='测试集适应度')

ax1.set_xlabel('优化迭代次数', fontsize=12)

ax1.set_ylabel('适应度(准确率)', fontsize=12)

ax1.set_title('优化算法收敛曲线', fontsize=14)

ax1.grid(True, linestyle='--', alpha=0.7)

# 设置x轴刻度为整数

ax1.xaxis.set_major_locator(plt.MaxNLocator(integer=True))

# 第二个y轴：参数变化

ax2 = ax1.twinx()

ax2.plot(iter_nums, self.history['alpha'], 'g-s', linewidth=1.5, label='Alpha参数')

ax2.set_ylabel('Alpha参数值', fontsize=12)

# 合并图例

lines1, labels1 = ax1.get_legend_handles_labels()

lines2, labels2 = ax2.get_legend_handles_labels()

ax1.legend(lines1 + lines2, labels1 + labels2, loc='best', fontsize=10)

plt.tight_layout()

plt.savefig(save_path)

print(f"收敛曲线已保存到: {save_path}")

# 绘制参数变化曲线

plt.figure(figsize=(10, 8))

plt.subplot(2, 1, 1)

plt.plot(iter_nums, self.history['iterations'], 'm-o', linewidth=2)

plt.ylabel('迭代次数参数', fontsize=12)

plt.title('模型参数优化过程', fontsize=14)

plt.grid(True, linestyle='--', alpha=0.7)

plt.xaxis.set_major_locator(plt.MaxNLocator(integer=True))

plt.subplot(2, 1, 2)

plt.plot(iter_nums, self.history['learning_rate'], 'c-o', linewidth=2)

plt.xlabel('优化迭代次数', fontsize=12)

plt.ylabel('学习率参数', fontsize=12)

plt.grid(True, linestyle='--', alpha=0.7)

plt.xaxis.set_major_locator(plt.MaxNLocator(integer=True))

plt.tight_layout()

plt.savefig(save_path.replace('.jpg', '_params.jpg'))

print(f"参数变化曲线已保存到: {save_path.replace('.jpg', '_params.jpg')}")

def main():

# 参数设置

parser = argparse.ArgumentParser(description="CNN-CatBoost-lstm-GSA-SSA振动信号分类")

## 数据路径

parser.add_argument("--save_dir", type=str,

default="D:\\python\\Classification\\results_LSTM-CNN-catboost\\features",

help="保存特征的目录")

parser.add_argument("--test_data_path", type=str,

default="D:\\桌面\\光纤传感信号识别\\Phi-OTDR_dataset_and_codes\\das_data\\test\\",

help="测试数据路径")

parser.add_argument("--test_label_path", type=str,

default="D:\\桌面\\光纤传感信号识别\\Phi-OTDR_dataset_and_codes\\das_data\\test\\label.txt",

help="测试标签路径")

parser.add_argument("--max_iterations", type=int, default=5,

help="最大优化迭代次数")

parser.add_argument("--cv_folds", type=int, default=3,

help="交叉验证折数")

args = parser.parse_args()

# 创建保存目录

os.makedirs(args.save_dir, exist_ok=True)

# 检查GPU可用性

device = torch.device("cuda:0" if torch.cuda.is_available() else "cpu")

print(f"使用设备: {device}")

# 加载特征

print("加载特征...")

train_features, train_labels, test_features, test_labels = load_features(args.save_dir)

# 特征标准化

print("进行特征标准化...")

scaler = StandardScaler()

train_features_scaled = scaler.fit_transform(train_features)

test_features_scaled = scaler.transform(test_features)

print(f"训练特征形状: {train_features_scaled.shape}")

print(f"测试特征形状: {test_features_scaled.shape}")

# 创建CatBoost基础参数

base_params = {

'iterations': 200,

'learning_rate': 0.1,

'depth': 6,

'l2_leaf_reg': 3,

'random_strength': 1,

'task_type': 'GPU' if torch.cuda.is_available() else 'CPU',

'devices': '0:0' if torch.cuda.is_available() else None,

'verbose': 200

}

# 基线模型评估

print("\n=== 基线模型评估 ===")

base_model = CatBoostClassifier(**base_params)

base_model.fit(train_features_scaled, train_labels)

base_preds = base_model.predict(test_features_scaled)

base_accuracy = accuracy_score(test_labels, base_preds)

print(f"基线模型准确率: {base_accuracy:.4f}")

# 打印分类报告

print("\n基线模型分类报告:")

print(classification_report(test_labels, base_preds))

# 使用CombinedOptimizer进行优化

print("\n=== 开始组合优化过程 ===")

optimizer = CombinedOptimizer(

base_params,

train_features_scaled,

train_labels,

test_features_scaled,

test_labels,

cv_folds=args.cv_folds

)

best_model, best_weights, best_params = optimizer.run(max_iterations=args.max_iterations)

# 最终评估

print("\n=== 最终模型评估 ===")

# 使用最佳模型和权重进行预测

weighted_test_features = test_features_scaled * best_weights

final_preds = best_model.predict(weighted_test_features)

final_accuracy = accuracy_score(test_labels, final_preds)

print(f"最终模型准确率: {final_accuracy:.4f}")

print("\n最终模型分类报告:")

print(classification_report(test_labels, final_preds))

# 计算提升幅度

improvement = (final_accuracy - base_accuracy) * 100

print(f"\n优化提升: {improvement:.2f}%")

# 显示最优模型参数

print("\n最优模型参数:")

for key, value in best_params.items():

print(f" {key}: {value}")

# 保存CatBoost模型和最优权重

final_model_info = {

'catboost': best_model,

'optimal_weights': best_weights,

'best_params': best_params,

'scaler': scaler,

'base_accuracy': base_accuracy,

'final_accuracy': final_accuracy,

'history': optimizer.history

}

joblib.dump(

final_model_info,

os.path.join(args.save_dir, "cnn_catboost-lstm-GSA-SSA2.0_model_combined.pkl")

)

print(

f"\nCatBoost模型和最优权重已保存到: {os.path.join(args.save_dir, 'cnn_catboost-lstm-GSA-SSA2.0_model_combined.pkl')}")

# 绘制混淆矩阵和特征权重分布

conf_matrix = confusion_matrix(test_labels, final_preds)

draw_confusion_matrix(conf_matrix, os.path.join(args.save_dir, "confusion_matrix_lstm-CNN-CatBoost-GSA-SSA2.0.jpg"))

# 提取特征重要性

feature_importances = best_model.get_feature_importance()

plot_feature_importance(

best_weights,

feature_importances,

os.path.join(args.save_dir, "feature_weights_lstm-GSA-SSA2.0.jpg")

)

# 保存优化结果摘要

with open(os.path.join(args.save_dir, "optimization_summary.txt"), "w") as f:

f.write("=== 优化结果摘要 ===\n")

f.write(f"基线模型准确率: {base_accuracy:.4f}\n")

f.write(f"优化后准确率: {final_accuracy:.4f}\n")

f.write(f"提升幅度: {improvement:.2f}%\n\n")

f.write("最优参数:\n")

for key, value in best_params.items():

f.write(f" {key}: {value}\n")

f.write("\n优化历史:\n")

for i in range(len(optimizer.history['iteration'])):

f.write(f"迭代 {optimizer.history['iteration'][i]}: ")

f.write(f"CV适应度={optimizer.history['cv_accuracy'][i]:.4f}, ")

f.write(f"测试适应度={optimizer.history['test_accuracy'][i]:.4f}, ")

f.write(f"Alpha={optimizer.history['alpha'][i]:.4f}, ")

f.write(f"迭代次数={optimizer.history['iterations'][i]}, ")

f.write(f"学习率={optimizer.history['learning_rate'][i]:.4f}\n")

print(f"优化结果摘要已保存到: {os.path.join(args.save_dir, 'optimization_GSA-SSA2.0.txt')}")

if __name__ == "__main__":

main()
